# The GLP-1 medicines semaglutide and tirzepatide do not alter disease-related pathology, behaviour or cognitive function in 5XFAD and APP/PS1 mice

**DOI:** 10.1016/j.molmet.2024.102019

**Published:** 2024-08-30

**Authors:** Leticia Forny Germano, Jacqueline A. Koehler, Laurie L. Baggio, Fiona Cui, Chi Kin Wong, Nikolaj Rittig, Xiemin Cao, Dianne Matthews, Daniel J. Drucker

**Affiliations:** Lunenfeld-Tanenbaum Research Institute, Mt. Sinai Hospital, Toronto, M5G1X5, Canada

**Keywords:** Diabetes, Obesity, Alzheimer's, Inflammation, Glucagon-like peptides, Neurodegeneration, GLP-1

## Abstract

**Objective:**

The development of glucagon-like peptide-1 receptor (GLP-1R) agonists for the treatment of type 2 diabetes and obesity has been accompanied by evidence for anti-inflammatory and cytoprotective actions in the heart, blood vessels, kidney, and brain. Whether GLP-1R agonists might be useful clinically for attenuating deterioration of cognitive dysfunction and reducing the progression of Alzheimer's disease remains uncertain.

**Methods:**

Here we evaluated the actions of semaglutide and tirzepatide, clinically distinct GLP-1 medicines, in two mouse models of neurodegeneration.

**Results:**

Semaglutide reduced body weight and improved glucose tolerance in 12-month-old male and female 5XFAD and APP/PS1 mice, consistent with pharmacological engagement of the GLP-1R. Nevertheless, amyloid plaque density was not different in the cerebral cortex, hippocampus, or subiculum of semaglutide-treated 12-month-old 5XFAD and APP/PS1 mice. IBA1 and GFAP expression were increased in the hippocampus of 5XFAD and APP/PS1 mice but were not reduced by semaglutide. Moreover, parameters of neurobehavioral and cognitive function evaluated using Open Field testing or the Morris water maze were not improved following treatment with semaglutide. To explore whether incretin therapies might be more effective in younger mice, we studied semaglutide and tirzepatide action in 6-month-old male and female 5XFAD mice. Neither semaglutide nor tirzepatide modified the extent of plaque accumulation, hippocampal IBA1+ or GFAP+ cells, or parameters of neurobehavioral testing, despite improving glucose tolerance and reducing body weight. mRNA biomarkers of inflammation and neurodegeneration were increased in the hippocampus of male and female 5XFAD mice but were not reduced after treatment with semaglutide or tirzepatide.

**Conclusions:**

Collectively, these findings reveal preservation of the metabolic actions of two GLP-1 medicines, semaglutide and tirzepatide, yet inability to detect improvement in structural and functional parameters of neurodegeneration in two mouse models of Alzheimer's disease.

## Highlights

• Semaglutide and tirzepatide produce weight loss in mice with neurodegeneration.
• Semaglutide and tirzepatide reduce glycemia in mice with neurodegeneration.
• Neither semaglutide nor tirzepatide improve cognition or behavioral testing.
• Neither semaglutide nor tirzepatide reduce plaque burden or neurinflammation.

## Graphical abstract

**Figure:**
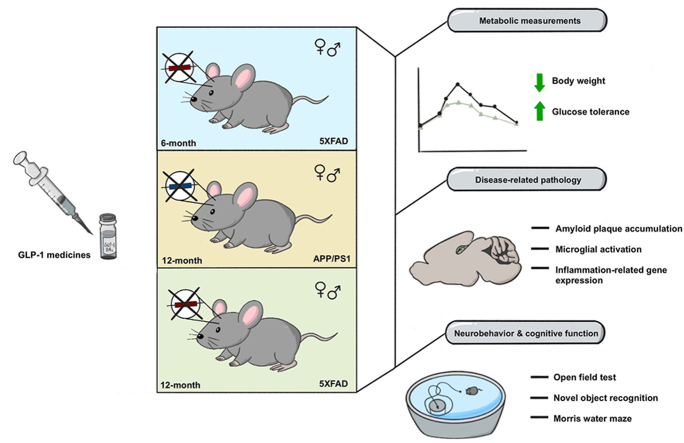

## 1 Introduction

Glucagon-like peptide-1 (GLP-1) is produced predominantly in gut endocrine cells, and circulates at low levels, acting as an incretin hormone to facilitate meal-related insulin secretion. GLP-1 also inhibits glucagon secretion and gastric emptying, actions contributing to its glucose-lowering effect [1]. Pharmacological doses of GLP-1 reduce food intake in animals and humans, enabling weight loss with chronic administration. Based on these actions, multiple glucagon-like peptide-1 receptor (GLP-1R) agonists (GLP-1RAs) have been developed for the treatment of type 2 diabetes (T2D); three agents, liraglutide, semaglutide, and tirzepatide, have been approved for weight loss in people living with overweight and related complications or obesity [2,3].

The GLP-1R is widely distributed beyond the pancreatic islets, and is expressed in the lung, heart, blood vessels, kidney, immune system, and in multiple regions of the brain [4]. GLP-1RAs reduce rates of myocardial infarction, stroke, and cardiovascular death in people with T2D, as well as in subjects living with overweight or obesity and pre-existing cardiovascular disease [2]. GLP-1RAs also improve functional outcomes in subjects with heart failure and preserved ejection fraction [5]. Intriguingly, GLP-1RAs also reduce liver inflammation in people with metabolic liver disease [6] and reduce the rates of chronic kidney disease in people with T2D [7]. Collectively, these therapeutic actions highlight the potential value of GLP-1-based medicines beyond the control of glucose and body weight [8].

GLP-1 is also expressed in the brain, predominantly in the brainstem, and transported throughout the central nervous system (CNS), interacting with widely distributed GLP-1Rs in multiple brain regions [9]. Beyond the control of appetite, GLP-1 induces aversive signals in animals and humans, with nausea and vomiting representing commonly reported side effects encountered with GLP-1 medicines in the clinic. Considerable data also support a potential neuroprotective effect of GLP-1RAs in the setting of experimental brain injury [10]. Moreover, multiple GLP-1RAs reduce inflammation and exert neuroprotective actions in mouse models of neurodegenerative disorders [11].

Accumulating evidence from preclinical studies, clinical trials, and real-world data support the contention that GLP-1RAs attenuate experimental brain injury and reduce the severity of neurodegeneration [11,12]. Hippocampal over-expression of GLP-1 in rats enhanced learning and memory, and activation of GLP-1R signaling in mice attenuated the severity of experimental brain injury, whereas *Glp1r*^−/−^ mice exhibited enhanced seizure severity and neuronal injury after kainate administration [10]. Over the past two decades, multiple GLP-1RAs have shown therapeutic benefit in mouse models of Alzheimer's disease, reducing the extent of amyloid-related pathology including plaque accumulation, improving results on behavioral and cognition testing, and decreasing neuroinflammation [11,12].

Complementary evidence supporting the potential for GLP-1RAs to modify the progression of neurodegenerative disorders stems from analysis of clinical trial data. A 12 week trial of 50 subjects with T2D treated with liraglutide revealed improvements in cognitive function, memory, and attention; findings independent of changes in blood pressure, glucose control, or body weight [13]. Similar outcomes, principally an increase in memory scores, were observed in 40 metformin-treated subjects with obesity and either T2D or prediabetes, randomized to receive liraglutide or placebo, and studied after achievement of ∼7% weight loss over ∼4 months [14]. Subjects with T2D with risk factors for or established cardiovascular disease and randomized to receive dulaglutide in the REWIND trial exhibited reduced rates of cognitive impairment over time, as assessed by serial analysis of test performance using the Montreal Cognitive Assessment and Digit Symbol Substitution tests [15]. In contrast, other studies have yielded mixed results. The Evaluation of Liraglutide in the treatment of Alzheimer's Disease (ELAD) trial failed to show a significant change in the primary endpoint of cerebral glucose metabolic rate after 12 months in people with mild to moderate Alzheimer's dementia treated with liraglutide. Nevertheless, liraglutide-treated subjects exhibited reduced brain shrinkage and improved scores on evaluation of cognitive function, a prespecified secondary endpoint [16]. Finally, data from cardiovascular outcome trials, together with real-world registry data, demonstrated reduced rates of new dementia diagnoses in people with T2D treated with GLP-1RAs [17,18].

To identify potential mechanisms linking the actions of GLP-1RAs to neuroprotection, we studied the effects of semaglutide, a GLP-1RA, and tirzepatide, a glucose-dependent insulinotropic polypeptide receptor (GIPR)-GLP-1R co-agonist, in the 5XFAD and APP/PS1 transgenic murine models of neurodegeneration. Unexpectedly, we did not detect beneficial effects of GLP-1 medicines on behavior, ionized calcium-binding adapter molecule 1 (IBA1) or Glial fibrillary acidic protein (GFAP) activation, CNS histology and amyloid plaque density, or gene expression profiles assessing biomarkers of inflammation or neurodegeneration.

## 2 Materials and methods

### 2.1 Animals

All procedures involving animals were performed in compliance with the Animals for Research Act of Ontario and the Guidelines of the Canadian Council on Animal Care. The Toronto Centre for Phenogenomics (TCP) Animal Care Committee reviewed and approved all procedures conducted on animals at the facility. Mice were housed up to five per cage on a 12-h light/dark cycle (7 am/7 pm) at 23C, with *ad libitum* access to water and regular diet (18% kcal from fat, Teklad 2918, Inotiv). APP/PS1: B6.Cg-Tg(APPswe, PSEN1dE9)85Dbo/Mmjax (Stock number 005864) and 5XFAD: B6.Cg-Tg(APPSwFlLon, PSEN1∗M146L∗L286V)6799Vas/Mmjax (Stock number 008730) male mice were obtained from Jackson Laboratories and bred at TCP by crossing with C57BL/6J (TCP) females. After weaning, mice were housed with wildtype (WT) littermates until the conclusion of the studies. 5XFAD genotyping was based on the 5XFAD primer sequence: CGG GCC TCT TCG CTA TTA C (Mutant-R 27367) and ACC CCC ATG TCA GAG TTC CT (Common 37598), and WT mice with primer sequence: TAT ACA ACC TTG GGG GAT GG (WT-R 37599) and ACC CCC ATG TCA GAG TTC CT (Common 37598). APP/PS1 genotyping was determined using the heterozygote primer sequence: ATGGTAGAGTAAGCGAGAACACG (Mutant-F 42433) and homozygote primer sequence: GGATCTCTGAGGGGTCCAGT (Common 42432). For WT mice, the primer sequence was GTGTGATCCATTCCATCAGC (WT-F 42431) and GGATCTCTGAGGGGTCCAGT (Common 42432). All genotyping primer sequences were obtained from the Jackson Laboratory.

### 2.2 Glucose tolerance tests and body composition measurements

Mice were fasted for 5 h prior to glucose tolerance testing. Glucose (1.5 g glucose per kg of body weight) was administered via the intraperitoneal (i.p.) route. Blood was collected from the tail vein into heparinized tubes at time 0- and 15-minutes following glucose administration; blood glucose levels were measured with a Contour handheld glucometer (Accu-Chek Aviva Roche) at 0, 15 30, 45, 60, and 120 min post-glucose injection. Body composition was assessed using a mouse whole-body Echo MRI system (Echo Medical Systems).

### 2.3 Drug treatments

All drugs were prepared as stock solutions and diluted daily to the final concentration before use. Dosing of semaglutide and tirzepatide was increased progressively (Supplementary Figures 1 and 5) to minimize aversive responses. For mice 6 months of age, the treatment period was 49 days (7 wks) and for older mice (12 months old), the treatment period was 42 days (6 wks). Initial body weights (g) of 12-month-old transgenic mice vs WT littermate controls were as follows: 5XFAD males 32.2 ± 4.1 vs WT 36.6 ± 3.8, 5XFAD females 23.5 ± 2.5 vs WT 28.9 ± 3.2, APP/PS1 males 43.8 ± 5.8 vs WT 46.7 ± 5.9, APP/PS1 females 31.7 ± 4.7 vs WT 39.3 ± 7.1. Initial body weights (g) of 6-month-old 5XFAD mice vs WT littermate controls were as follows: 5XFAD males 29.1 ± 1.9 vs WT 30.7 ± 3.4, 5XFAD females 22.5 ± 2.6 vs WT 23.4 ± 1.5. Vehicle-treated mice were administered an equivalent volume of phosphate buffered saline (PBS). Mice were injected subcutaneously (s.c.) with semaglutide or tirzepatide once daily (Supplementary Figures 1A and 5A). For Semaglutide, an initial dose of 10 nmol/kg (41.1 ug/kg) was administered for 1 week, followed by 17 nmol/kg (69.93 ug/kg) for 1 week, and 25 nmol/kg (102.8 ug/kg) for the final weeks. Tirzepatide doses were 2.5 nmol/kg (12 ug/kg), 5 nmol/kg (24 ug/kg), and 10 nmol/kg (48 ug/kg), for weeks 1, 2, and 3–7, respectively.

### 2.4 Behavioral studies

Mice were acclimatized to the behavior room for a minimum of 1 h prior to all procedures to minimize stress. All tests were conducted at the TCP within the Lunenfeld-Tanenbaum Research Institute at Mount Sinai Hospital. For all behavioral tasks, 6- or 12–13-month-old male and female 5XFAD, APP/PS1, and WT littermates were studied.

### 2.5 Open field test

During open-field experiments, mice were placed in an open-field arena (43.5 cm^2^) within a sound attenuating chamber equipped with a dimmable LED light and activity monitor software (Med Associates Inc.). Prior to introducing the mouse into the arena, the lighting was verified to be close to 200 lux in the arena center. Data for locomotor activity, time at the center and periphery, distance traveled (cm), and speed (cm/s) were collected over a period of 20 min for each mouse using infrared beam arrays. The testing arena was sanitized with a 70% ethanol solution and dried prior to introducing the next mouse.

### 2.6 Novel object recognition test

The Novel Object Recognition test was performed in an acrylic arena (W: 41.25 cm, L: 41.25 cm, H: 31.25 cm) illuminated from 2 m above using a 60-W lamp. The arena and objects were cleaned with 70% ethanol between trials to prevent the build-up of olfactory cues. The test was conducted over 3 days devoted to habituation, familiarization, and a test day. On the day of habituation, mice were placed in an empty arena for 5 min to become familiarized with the apparatus and test room and then returned to their home cage. Twenty-four hours later, mice were placed back in the arena now containing 2 identical objects placed 5 cm away from the walls for 10 min for object familiarization. Twenty-four hours after familiarization, mice were placed in the arena with one familiar object and a novel object for 10 min as the test day. The objects were placed at the same location; however, the novel object was randomized between the 2 positions for each mouse and treatment group tested. Sessions were recorded and video tracking was assessed using EthoVision XT video tracking software (Noldus). Object exploration is scored whenever the distance between the mouse's nose and the object is less than 2 cm or when the object is touched while looking at it. Mouse object preference was calculated by the discrimination index which is defined by the total time exploring the novel object less the time exploring the familiar object over the total time spent exploring both objects [19]. Positive values indicate preference for the novel object.

### 2.7 Morris Water Maze

Training trials were performed using a hidden platform submerged at 1 cm below the water surface and positioned in the middle of the same quadrant, 20 cm from the wall of the tank. Four trials were performed each day for 4 consecutive days with an intersection time between 10 and 15 min. Starting points were randomized between quadrants and changed for each trial. Four visual cues were distributed in the center of each quadrant on the room wall. Each training trial was deemed complete when the mouse reached the platform (escape latency) or after 60 s, whichever occurred first. Mice failing to reach the platform were guided onto it. After each training trial, mice remained on the platform for 30 s. The probe trial was performed on day 5, 24 h after the completion of training. On this day, the platform was removed, and mice were allowed to swim for 60 s. All trials were monitored by a video camera set above the center of the pool and the trajectories were recorded with a video tracking software system (Ethovision XT by Noldus).

### 2.8 Perfusion and preparation of blood and tissue samples

5XFAD mice were euthanized at 6 and 12–13 months of age, and APP/PS1 mice at 12–13 months. Mice were anesthetized with i.p. Avertin 2% (10 ul/g) prior to trans-cardiac perfusion with ice-cold PBS. Cardiac blood was collected immediately before perfusion and centrifuged for 5 min at 4 °C at 14,500 RPM; plasma was stored at −80 °C. For all studies, brains were removed, and hemispheres separated along the midline. The right brain was dissected on ice for regions of interest and snap frozen for subsequent biochemical analyses. The contralateral hemisphere was drop-fixed in 10% formalin for 48 h. Two parasagittal sections (middle of the half hemisphere) were then cut for embedding in paraffin and sectioned at 5 μm.

### 2.9 Thioflavin S staining

For Thioflavin S staining to detect amyloid plaques, 4 sections per mouse were heated to 50 °C for 10 min, immersed in xylene 100% (2 × 5 min), and dehydrated in a graded series of ethanol (100%, 95%, 70%, 50%; 1 × 3 min each). Sections were then incubated in 0.5% Thioflavin S (in 50% ethanol, Sigma–Aldrich, Cat# T1892) for 10 min and washed with distilled water (1 × 3 min) and 1× PBS (1 × 5 min), and then immersed in 50% ethyl alcohol (1 × 5 min). Sections were then washed in 1X PBS (1 × 10 min) and mounted with Prolong (Invitrogen, Cat# P10144). Slides were imaged on a Zeiss Axio Z1 Scanner with Zen blue software using a 20X objective. Images were exported to Visiopharm software to quantify fluorescent intensity, area, and positivity/number.

### 2.10 Thioflavin S quantitative analysis

Amyloid burden was measured by quantification of the total number, size, and intensity of Aβ plaques. Aβ plaques (expressed in area units, μm^2^) in the entire cortical and hippocampal areas were analyzed in individual sagittal sections from the cerebral cortex, hippocampus, and subiculum. Regions of interest were delineated manually using Visiopharm software (Visiopharm, Hoersholm, Denmark). For plaque intensity, a minimum threshold was determined for background exclusion and maintained throughout the analysis per timeframe for uniformity. The total number of amyloid plaques and their area was obtained automatically by Visiopharm software and visually verified after analysis. Amyloid plaques, area, and intensity were quantified by Visiopharm software and normalized to the total area of the hippocampus, subiculum, and cortex. Quantitative comparisons between groups were carried out on comparable sections that were processed at the same time using the same batch of solutions.

### 2.11 IBA1 and GFAP quantitative analyses

Microglial activation was measured by quantifying the total number of IBA1-positive cells, and reactive astrocytes were evaluated by counting the number of GFAP-positive cells. Sections were immersed in xylene 100% (3 × 5 min) and dehydrated in a graded series of ethanol (100%, 95%; 2 × 10 min each) and water (2 × 5 min). Antigen retrieval was performed by heating at 95 °C for 20 min in 1X TE buffer at pH 9 (10 mM Tris, 1 mM EDTA tetrasodium, 0.05% Tween 20) and rinsed for 15 min in water. Sections were then blocked in 100 μl blocking buffer (10% goat serum and 0.1% Triton™ X-100 in tris-buffered saline with Tween [TBST]) for 30 min at room temperature and incubated overnight at 4 °C with 100 μl of IBA1 primary antibody (Ab, 1:1000, Anti-IBA1 Rabbit, Cat# 019-19741, Fujifilm) or GFAP primary Ab (1:1000, Anti-GFAP Rat, Cat# 13-0300, Invitrogen) diluted in TBST containing 0.2% goat serum. Slides were then washed with TBST (3 × 5 min) and incubated for 1 h at room temperature with 100 μl of secondary Ab (1:400, Goat anti-Rabbit IgG (H + L) Cross-Adsorbed Secondary Antibody Alexa Fluor® 488 conjugate Cat# A-11008, Invitrogen, or 1:400, Goat anti-Rat IgG (H + L) Cross-Adsorbed Secondary Antibody Alexa Fluor® Plus 594 Cat# A-48264, Invitrogen) diluted in PBS containing 4′,6-diamidino-2-phenylindole (1:1000). Sections were then washed in TBST (3 × 5 min) and mounted with Prolong (Invitrogen, Cat# P10144). Activated microglia, expressed as the number of IBA1-positive cells per unit area (μm^2^), or reactive astrocytes (astrogliosis), expressed as the number of GFAP-positive cells per unit area (μm^2^), were analyzed within the hippocampus and subiculum of sagittal brain sections. Regions of interest were drawn manually using Visiopharm software (Visiopharm, Hoersholm, Denmark) with the Allen Brain Atlas as a reference (Allen Institute for Brain Science). Positive cells were selected on the basis of both minimum intensity and size thresholds; thresholds were maintained throughout analyses for all samples to ensure uniformity. The number of activated microglial cells was obtained using the Visiopharm software and verified manually after blinded analysis.

### 2.12 Real-time qPCR for gene expression analysis

Hippocampal RNA was isolated using Tri Reagent (TRIzol, MRC) and the TissueLyser II (Qiagen), according to manufacturer instructions, and precipitated with ethanol. Total RNA (1 μg) was treated with DNase1 (Thermo Fisher Scientific, EN0521) and cDNA was synthesized using Superscript III (Thermo Fisher Scientific, 18080044) and random primers (Invitrogen, 58875). Quantitative PCR was performed in 384-well plates with TaqMan Fast Advanced Master Mix (Thermo Fisher) and Taqman probes (Supplementary Table 1) and run on a QuantStudio 5 system (Thermo Fisher). Relative expression was calculated by the 2^−ΔΔCT^ method using *Ppia* as the reference gene.

### 2.13 Data analyses and statistics

Behavioral, biochemical, and immunohistology data were analyzed using The Shapiro–Wilk test to validate the normality of the distribution of the samples and indicate the appropriate test (i.e. parametric or non-parametric test). Student's t-test, Mann–Whitney *U* test, one-way ANOVA, two-way or mixed effect ANOVA were performed using GraphPad Prism Version 9 (GraphPad). Sidak's and Tukey's post-hoc tests were used to identify biologically relevant interactions from the ANOVA. Outliers were excluded when detected by Rout test with Q = 0.05% on Thioflavin S and gene expression data. Animals that did not move during the open field test or presented health concerns (such as eye or locomotion issues), were excluded. Data are represented as means and standard deviation (SD), or error (SEM) of the mean as indicated in the figure legends. Each mouse is represented by an individual data point on scattered plots as indicated in the figure legends.

## 3 Results

### 3.1 Semaglutide induces weight loss and improves glucose tolerance in 5XFAD and APP/PS1 mice

**Figure 1:**
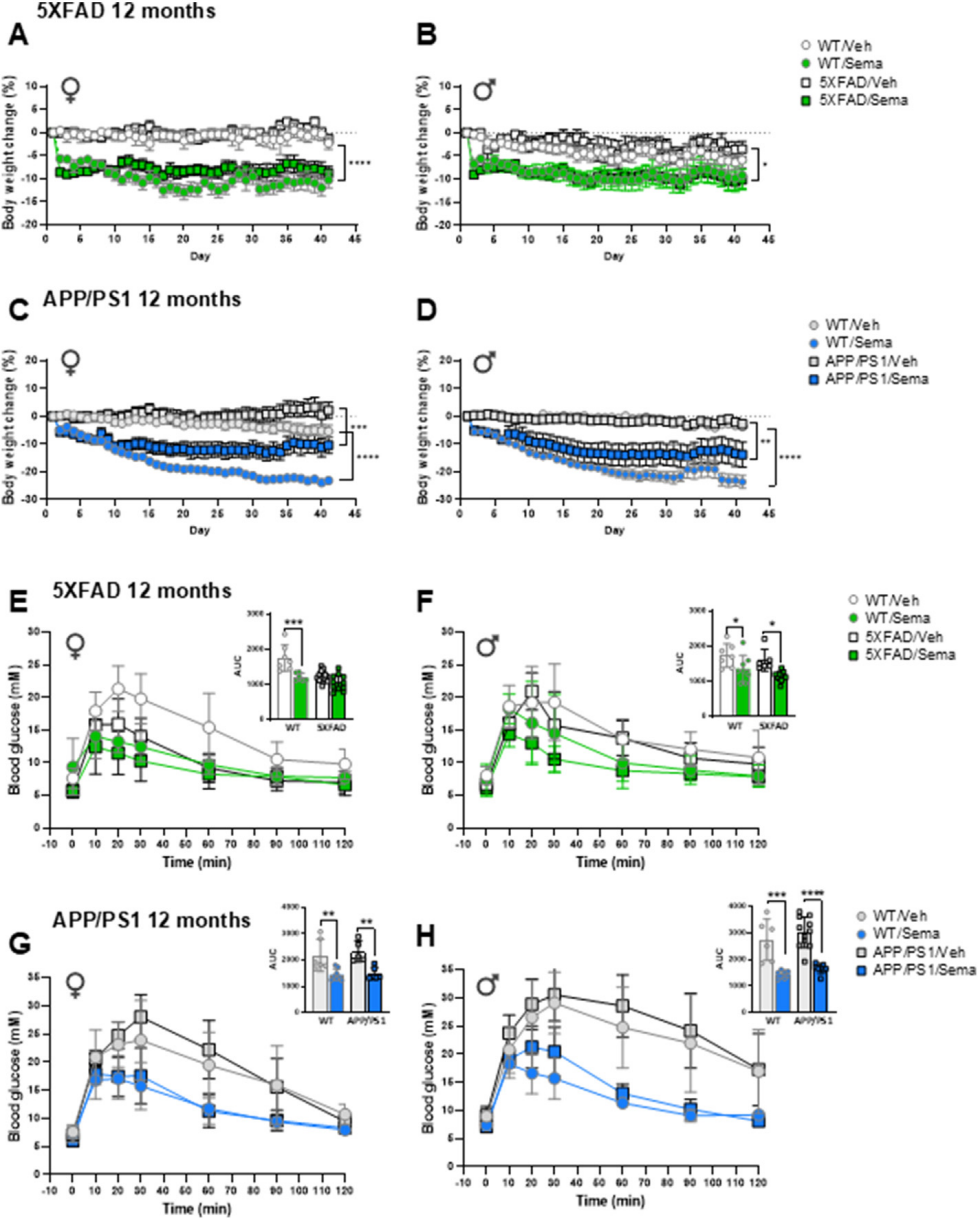
**Semaglutide treatment induces body weight loss and improves glucose tolerance in 12-month-old 5XFAD and APP/PS1 mice.** Daily percentage of body weight change from baseline of regular chow diet-fed 12-month-old 5XFAD (A, B), APP/PS1 (C, D) and wildtype matched female (♀) and male (♂) mice as indicated. Mice were treated with weekly incremental doses of semaglutide (10, 17, 25 nmol/kg) as shown in Supplemental Figure 1A. Glucose excursions following an i.p. glucose challenge in 12-month-old 5XFAD (E, F), APP/PS1 (G, H) and wildtype matched female (♀) and male (♂) mice treated with semaglutide (25 nmol/kg) or vehicle (saline). Inset graphs represent the Area Under the Curve (AUC) of the blood glucose excursions. AUC data for AD and E-H were analyzed by 2-way ANOVA with Sidak's post-hoc test for comparison of WT or transgenic-vehicle vs. semaglutide-treated mice. ∗*p* < 0.05, ∗∗*p* < 0.01, ∗∗∗*p* < 0.001, ∗∗∗∗*p* < 0.0001 vehicle vs. semaglutide-treated mice. Data are represented as means ± SEM (A–D) or means ± SD (E-H, and AUCs). n = 5–13 in each group of vehicle-(Veh) or semaglutide-(Sema) treated mice.

Weekly up-titration of once daily semaglutide administration to a final dose of 25 nmol/kg/day (Supplementary Figure 1A) lowered body weight in 12-month-old male and female 5XFAD and APP/PS1 mice (Figure 1A–D), two commonly studied mouse models of neurodegeneration. Semaglutide-treated 5XFAD mice exhibited less weight loss relative to the magnitude of weight loss observed in APP/PS1 mice, which may partially be due to differences in starting body weights (5XFAD females 23.5 g ± 2.5 vs APP/PS1 females 31.7 g ± 4.7; 5XFAD males 32.2 g ± 4.1 vs APP/PS1 males 43.8 g ± 5.8) (Figure 1A–D; Supplementary Figure 1B-M). Moreover, WT control mice lost more weight than APP/PS1 mice following semaglutide treatment (Figure 1C,D). Weight loss was associated with a corresponding reduction in fat mass in both male and female APP/PS1 mice and an increase in lean mass in APP/PS1 but not in 5XFAD mice treated with semaglutide (Supplementary Figure 1N-U). Semaglutide improved glucose tolerance in male, but not in female, 5XFAD mice (Figure 1E,F), consistent with the lower baseline glycemic excursions in female vs. male 5XFAD mice (Figure 1E,F). Semaglutide also improved glucose tolerance in both male and female APP/PS1 mice (Figure 1G,H).

### 3.2 Thioflavin S aggregates are not altered in 12-month-old 5XFAD and APP/PS1 mice following semaglutide treatment

**Figure 2:**
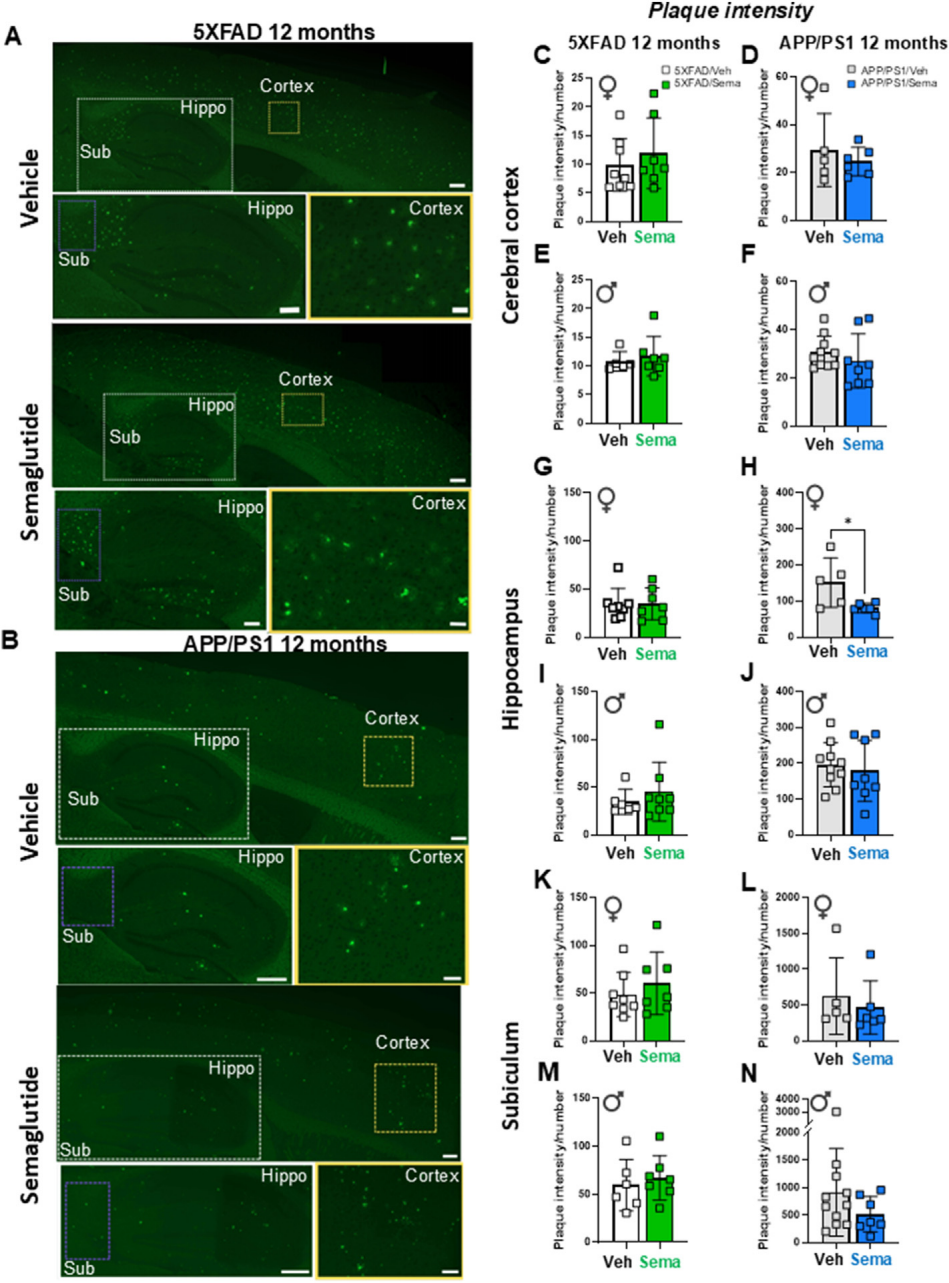
**Effect of semaglutide on amyloid beta content in 12-month-old 5XFAD and APP/PS1 mice.** Representative images of Thioflavin S staining of vehicle- and semaglutide (25 nmol/kg)-treated 5XFAD (A) and APP/PS1 (B) mice. Bottom panels in each image represent magnification of areas highlighted in the upper panels of the cerebral cortex (Cortex), hippocampus (Hippo) and subiculum (Sub) regions. Scale bars represent 100 μm (upper panels), 50 μm (lower panel for hippocampus and subiculum) and 10 μm (lower panel for cortex). Quantification of the plaque intensity in the cerebral cortex (C–F), hippocampus (G–J) and subiculum (K–N) after Thioflavin S staining in vehicle- or semaglutide (25 nmol/kg)-treated 12-month-old 5XFAD (left panels) and APP/PS1 (right panels) female (♀) and male (♂) mice. Data were analyzed by Mann–Whitney *U* test, or unpaired, two-tailed Student's t test. ∗*p* < 0.05 vehicle vs. semaglutide-treated mice. Data are represented as means ± SD. n = 5–11 in each group of vehicle (Veh)- or semaglutide (Sema)-treated mice.

To assess amyloid beta content, sagittal brain sections were stained with Thioflavin S (a fluorescent dye that binds to β amyloid), focusing on detection of plaques in the cerebral cortex and hippocampus. As the subiculum is the hippocampal region with the greatest accumulation of Aβ plaques [20,21], it was evaluated separately from the rest of the hippocampus. Representative images of Aβ plaque accumulation in the brains of 12-month-old 5XFAD and APP/PS1 mice are shown in Figure 2A,B. Semaglutide treatment did not consistently reduce plaque intensity, plaque number/surface area, or plaque area in the cerebral cortex, hippocampus, or subiculum of male or female 5XFAD or APP/PS1 mice (Figure 2CN; Supplementary Figure 2). Plaque intensity was lower in the hippocampus of semaglutide-treated female APP/PS1 mice (Figure 2H), but not different in male APP/PS1 mice, nor in male or female 5XFAD mice (Figure 2C-N).

### 3.3 Semaglutide does not reduce hippocampal microglial activation or reactive astroglia in 12-month-old 5XFAD and APP/PS1 mice

**Figure 3:**
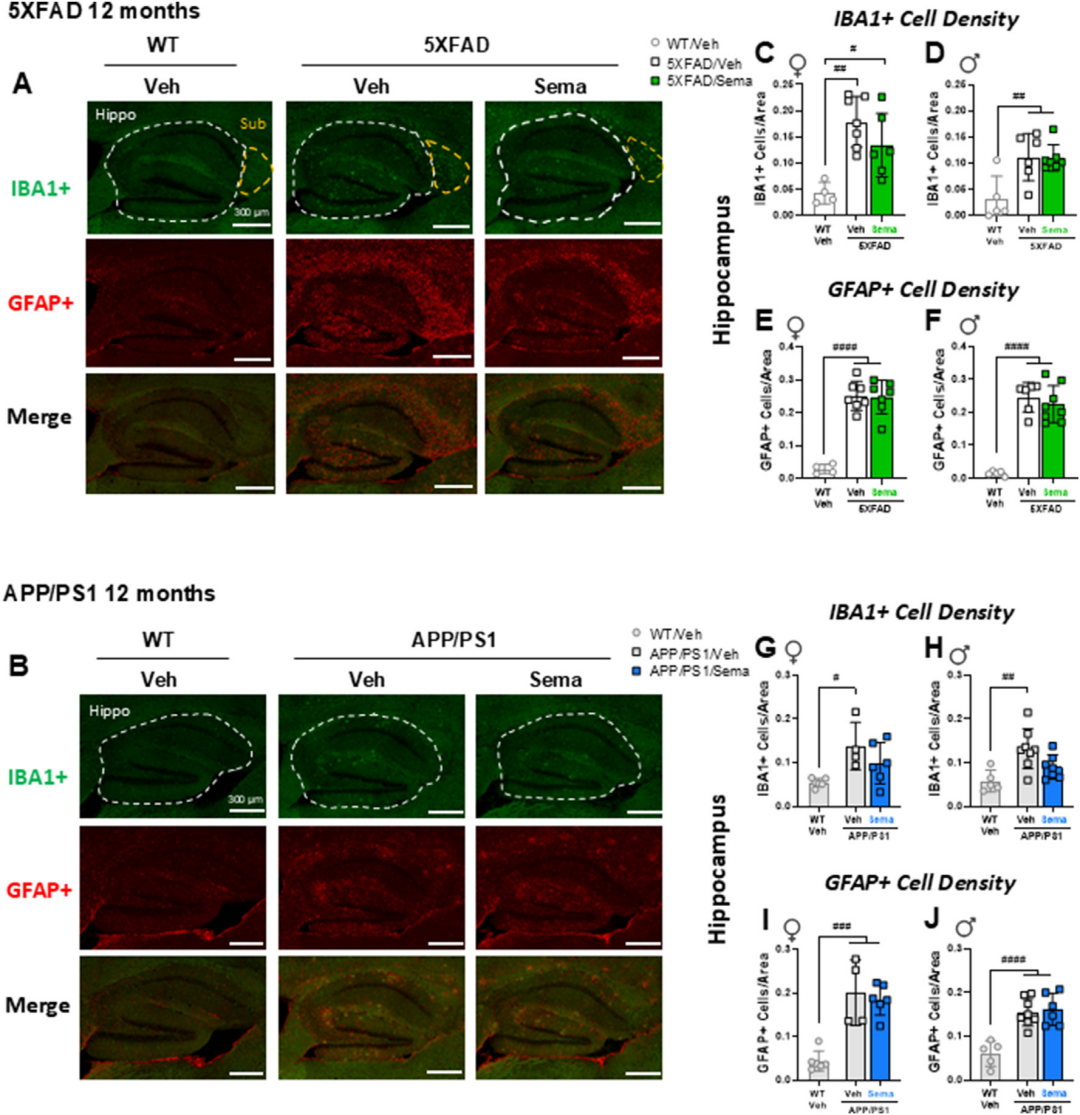
**Effect of semaglutide on activated microglia (IBA1+) and reactive astroglia (GFAP+) in 12-month-old 5XFAD and APP/PS1 mice.** Representative images of IBA1+ cells (green), GFAP+ cells (red), or cells co-stained with both IBA1 and GFAP antibodies (bottom panel) in the hippocampus (Hippo) or subiculum (Sub) of 12-month-old WT and vehicle- or semaglutide (25 nmol/kg)-treated 5XFAD (A) and APP/PS1 (B) mice. Scale bars represent 300 μm. Areas of interest are encircled by dashed lines. (C–J) Quantification of the number of IBA1+ cells per area (C, D) and the number of GFAP+ cells per area (E, F) in the hippocampus of 12-month-old female (♀) and male (♂) WT and 5XFAD mice. Quantification of the number of IBA1+ cells per area (G, H) and the number of GFAP+ cells per area (I, J) in the hippocampus of 12-month-old female (♀) and male (♂) WT and APP/PS1 mice. Data (C–J) were analyzed by 1-way ANOVA with Tukey's post-hoc test. #*p* < 0.05, ##*p* < 0.01 ###*p* < 0.001, ####*p* < 0.0001 WT vs. transgenic mice. Data are represented as means ± SD. n = 4–8 in each group of vehicle (Veh)- or semaglutide (Sema)-treated mice.

Activation of microglial cells, the resident macrophages of the 10.13039/100015097CNS, and the transition of astrocytes, a type of supporting glial cell in the 10.13039/100015097CNS, to a reactive phenotype (astrogliosis), have been implicated in the pathogenesis of neurodegenerative diseases. The extent of microglial activation, as measured by the density of IBA1-positive microglial cells, was increased in the hippocampus of both 5XFAD and APP/PS1 mice (Figure 3A–D, G-H), yet was not different in the hippocampus of 12-month-old male and female 5XFAD or APP/PS1 mice treated with semaglutide (Figure 3C–D, G-H). Similarly, the extent of astrogliosis, as measured by the density of GFAP-positive astrocytes, was increased in the hippocampus of 5XFAD and APP/PS1 mice, yet not different after treatment with semaglutide (Figure 3E–F, I-J).

### 3.4 Behaviour and cognition are not different in semaglutide-treated 5XFAD and APP/PS1 mice

**Figure 4:**
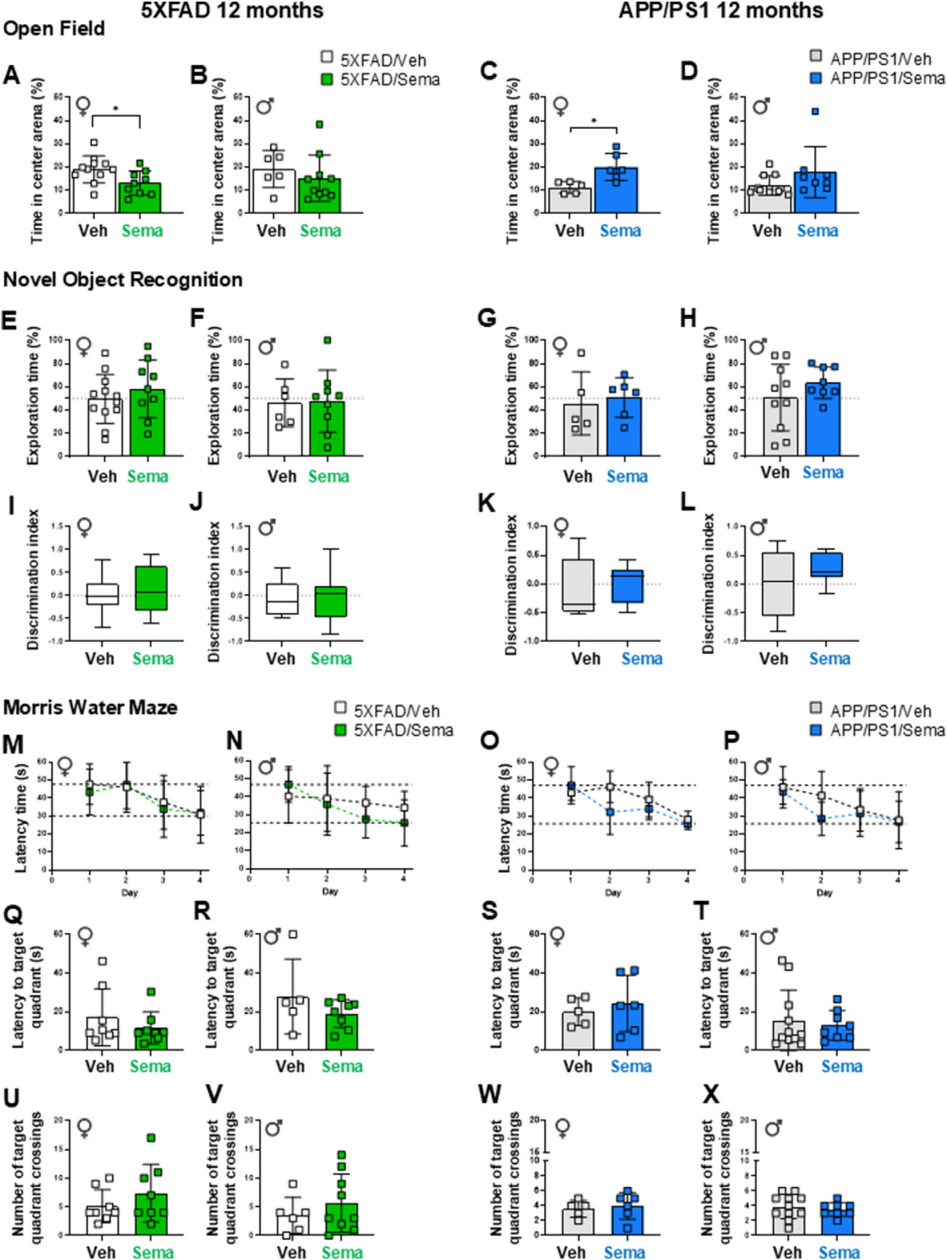
**Cognitive test performance of 12-month-old 5XFAD and APP/PS1 mice with semaglutide or vehicle treatment.** (A–D) **Open Field test.** The percentage of time spent in center area of the arena of vehicle- or semaglutide (25 nmol/kg)-treated 5XFAD (A, B) and APP/PS1 (C, D) female (♀) and male (♂) mice. (E–L) **Novel Object Recognition** test was performed to assess exploratory behavior and recognition memory. (E–H) The percentage of exploration time on the novel object of vehicle- or semaglutide (25 nmol/kg)-treated 5XFAD (E, F) and APP/PS1 (G, H) mice. (I–L) The discrimination index, defined by the time spent investigating the novel object over the total object exploration time, of vehicle- or semaglutide (25 nmol/kg)-treated 5XFAD (I, J) and APP/PS1 (K, L) mice. (M–P) **Morris Water Maze (Training days).** Daily latency time to reach the platform during the 4-day trial acquisition task (training) of vehicle- or semaglutide (25 nmol/kg)-treated 5XFAD (M, N) and APP/PS1 (O, P) female (♀) and male (♂) mice. (Q–X) **Morris Water Maze (Probe test day)**. Latency time to the target quadrant on Probe test day (Day 5) of vehicle- or semaglutide (25 nmol/kg)-treated 5XFAD (Q, R) and APP/PS1 (S, T) mice. The number of crossings on the target quadrant of vehicle- or semaglutide (25 nmol/kg)-treated 5XFAD (U, V) and APP/PS1 (W, X) mice. Data on (M–P) were analyzed by 2-way ANOVA with Sidak's post-hoc test comparison, and on (A-L, Q-X) by Mann–Whitney *U* test, or unpaired, two-tailed Student's t test. ∗*p* < 0.05 vehicle- vs. semaglutide-treated mice. Data are represented as means ± SD (A-H, M-X), or as box-and-whisker plots (I–L). Dashed lines in panels M–P indicate the time range (longest and shortest time) required to reach the platform over the 4-day training period. n = 5–11 in each group of vehicle (Veh)- or semaglutide (Sema)-treated mice.

We next used behavioral tests (Supplementary Figure 1A) to interrogate the impact of semaglutide administration on cognitive behavior. Open field testing assessed locomotor activity and anxiety-like behaviour, whereas Novel Object Recognition and Morris Water Maze (MWM) tasks assessed exploration, recognition, and spatial memory [20,22,23]. In the Open field test, distance travelled was similar between 12-month-old transgenic animals and their respective WT controls, regardless of treatment (Supplementary Figure 3A-D). In contrast, 5XFAD, but not APP/PS1, mice spent more time in the center of the arena (an indicator of reduced anxiety) than WT control mice (Supplementary Figure 3E-H). Semaglutide treatment did not consistently affect either of these parameters in 5XFAD or APP/PS1 mice (Figure 4A–D, Supplementary Figure 3 A-D). However, semaglutide treatment was associated with reduced time spent in the center area in female 5XFAD mice (Figure 4A), whereas female APP/PS1 mice spent more time in the center area (Figure 4C). In the Novel Object Recognition test, no differences in percent exploration time, or discrimination index were detected in semaglutide-treated male and female 5XFAD and APP/PS1 mice (Figure 4EL). Similarly, semaglutide had no consistent effect on these behaviors in WT control mice (Supplementary Figure 3I-P).

Analysis of MWM data revealed longer latency times to the platform (an indirect readout for learning) in female 5XFAD and APP/PS1 mice compared to WT control mice during the 4-day training period, irrespective of treatment (Supplementary Figure 4A-D, Figure 4M−P). On test day (Day 5), the latency time to the target quadrant remained higher in female APP/PS1 mice compared to WT mice (Supplementary Figure 4G), however semaglutide treatment did not affect latency times, or the number of target quadrant crossing events in 12-month-old male and female 5XFAD or APP/PS1 mice (Figure 4Q–X). Similarly, semaglutide did not modify these parameters in age- and sex-matched control mice (Supplementary Figure 4E-L). Moreover, total distance traveled, and swim speed were not different in semaglutide vs. vehicle-treated 5XFAD and APP/PS1 mice and were comparable to WT control mice (Supplementary Figure 4M-T).

### 3.5 Body weight and glycemic responses in 6-month-old 5XFAD mice treated with semaglutide or tirzepatide

**Figure 5:**
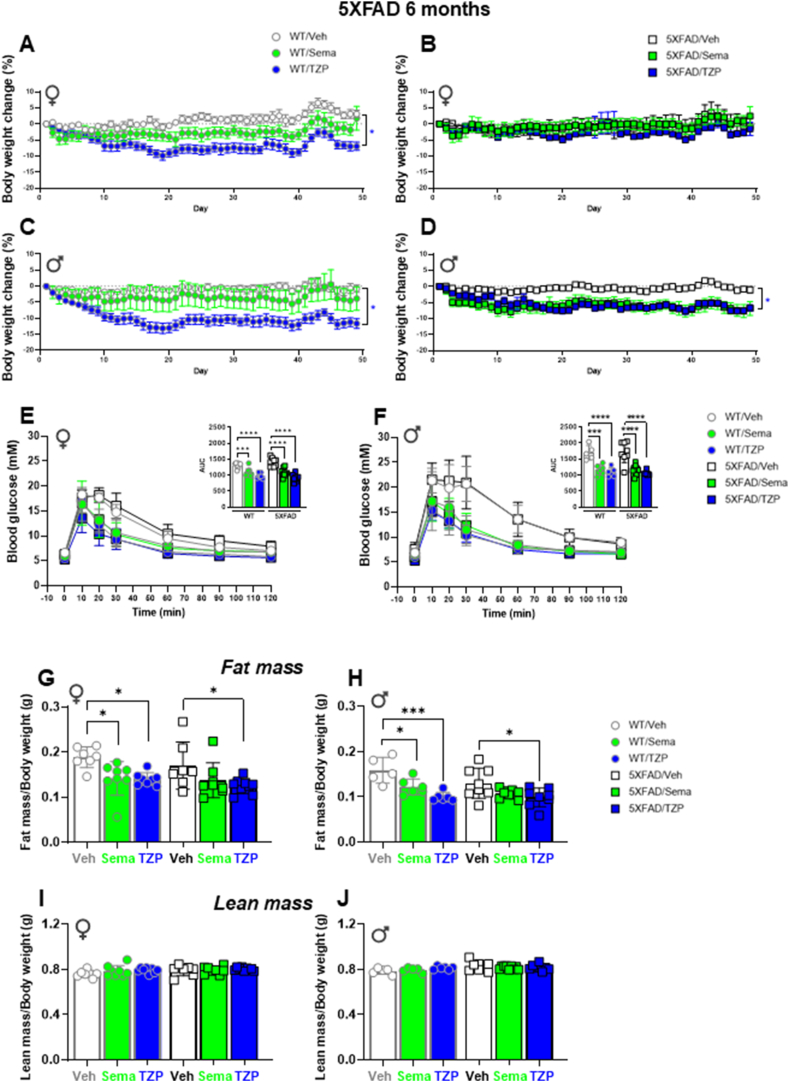
**Metabolic characterization of 6-month-old wildtype and 5XFAD mice treated with semaglutide or tirzepatide.** The percentage of body weight change from baseline of regular chow diet-fed 6-month-old female (♀) and male (♂) WT (A, C) and 5XFAD (B, D) mice. Mice were treated with weekly incremental doses of semaglutide (10, 17, 25 nmol/kg), or tirzepatide (2.5, 5, 10 nmol/kg) as indicated in Supplemental Figure 5. (E, F) Glucose excursions following an i.p. glucose challenge in regular chow diet-fed 6-month-old 5XFAD and WT-matched female (E) and male (F) mice treated with semaglutide (25 nmol/kg), tirzepatide (10 nmol/kg), or vehicle (saline). Inset graphs represent the Area Under the Curve (AUC) of the blood glucose excursions. (G–J) Body composition assessed by EchoMRI. Fat mass normalized by body weight of vehicle-, semaglutide (25 nmol/kg)-, or tirzepatide (10 nmol/kg)-treated 5XFAD and WT-matched female (G) and male (H) mice. Lean mass normalized by body weight of vehicle-, semaglutide (25 nmol/kg)-, or tirzepatide (10 nmol/kg)-treated 5XFAD and WT-matched female (I) and male (J) mice. Data were analyzed by 2-way ANOVA with Tukey's post-hoc test for comparison of vehicle vs. treatment. ∗*p* < 0.05, ∗∗∗*p* < 0.001, ∗∗∗∗*p* < 0.0001 vehicle vs. treatment. Data are represented as means ± SEM (A–D), or as means ± SD (E-J and AUCs). n = 5–10 in each group of vehicle (Veh)-, semaglutide (Sema)-, or tirzepatide (TZP)-treated mice.

As analyses in 12-month-old 5XFAD and APP/PS1 mice did not reveal semaglutide-associated improvements in CNS pathology, neurobehavior, or cognition, we hypothesized that GLP-1-based medicines might be more effective in younger animals, as has been demonstrated for the actions of GLP-1 on β-cell mass [24]. Accordingly, we analyzed the effects of semaglutide as well as the dual GLP-1R and GIPR co-agonist, tirzepatide in 6-month-old 5XFAD mice using an experimental regimen similar to that described for 12-month-old mice (Supplementary Figures 1A and 5). Weight loss was observed with both semaglutide and tirzepatide but was greater in tirzepatide-treated male and female WT control vs 5XFAD mice (Figure 5A–D). Both tirzepatide and semaglutide robustly improved glucose tolerance following i.p. glucose challenge (Figure 5E,F). Analysis of body composition revealed reductions in fat mass that mirrored changes in body weight, with no change in lean mass, in male and female 5XFAD mice treated with semaglutide or tirzepatide (Figure 5G–J).

### 3.6 Neither semaglutide nor tirzepatide alter amyloid beta content in 6-month-old 5XFAD mice

**Figure 6:**
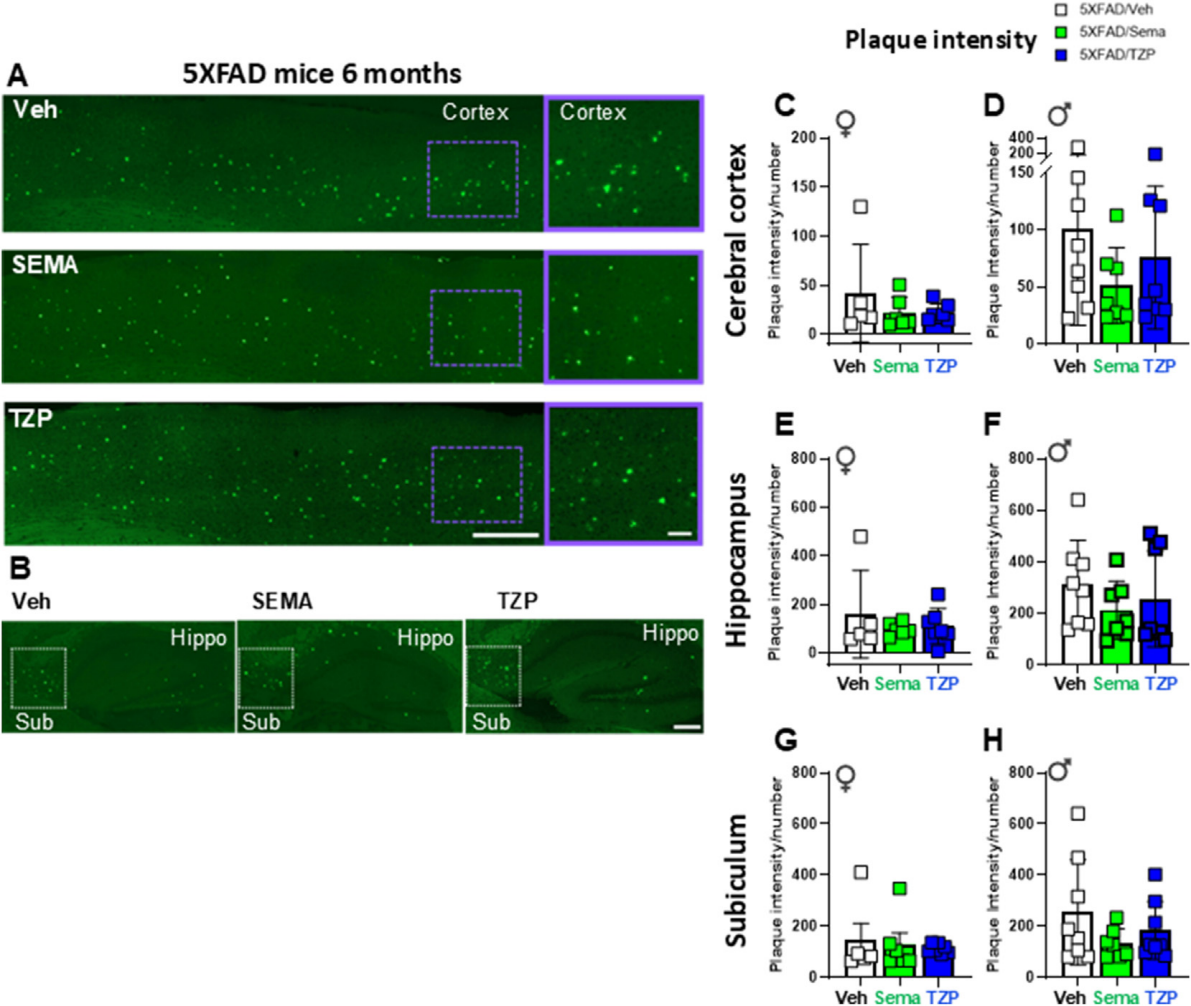
**Semaglutide and tirzepatide do not alter amyloid beta content in 6-month-old 5XFAD mice.** (A, B) Representative images of (A) the cerebral cortex (Cortex) and (B) the hippocampus (Hippo) and subiculum (Sub) brain regions of 5XFAD mice. Right panels in (A) represent magnification of the area of the cerebral cortex highlighted in the left panels. Scale bars in (A) represent 100 μm (left panels) and 10 μm (right panels); scale bar in (B) represents 50 μm. Quantification of plaque intensity in the cerebral cortex (C, D), hippocampus (E, F) and subiculum (G, H) after Thioflavin S staining in 6-month-old 5XFAD and WT-matched female (♀) and male (♂) mice treated with semaglutide (25 nmol/kg), tirzepatide (10 nmol/kg), or vehicle (saline). Data are represented as means ± SD. n = 5–11 in each group of vehicle (Veh)-, semaglutide (Sema)-, or tirzepatide (TZP)-treated mice.

Amyloid content was analyzed by plaque intensity, number of plaques, and plaque area. Amyloid intensity and plaque number were not different in the cerebral cortex, hippocampus, or subiculum of semaglutide- or tirzepatide-treated 6-month-old male or female 5XFAD mice vs vehicle-treated controls (Figure 6 and Supplementary Figure 6). Unexpectedly, relative plaque area was increased in the cerebral cortex of tirzepatide- vs vehicle-treated 5XFAD male mice (Supplementary Figure 6H).

### 3.7 Tirzepatide improves novel object recognition task performance in 6-month-old male 5XFAD mice

**Figure 7:**
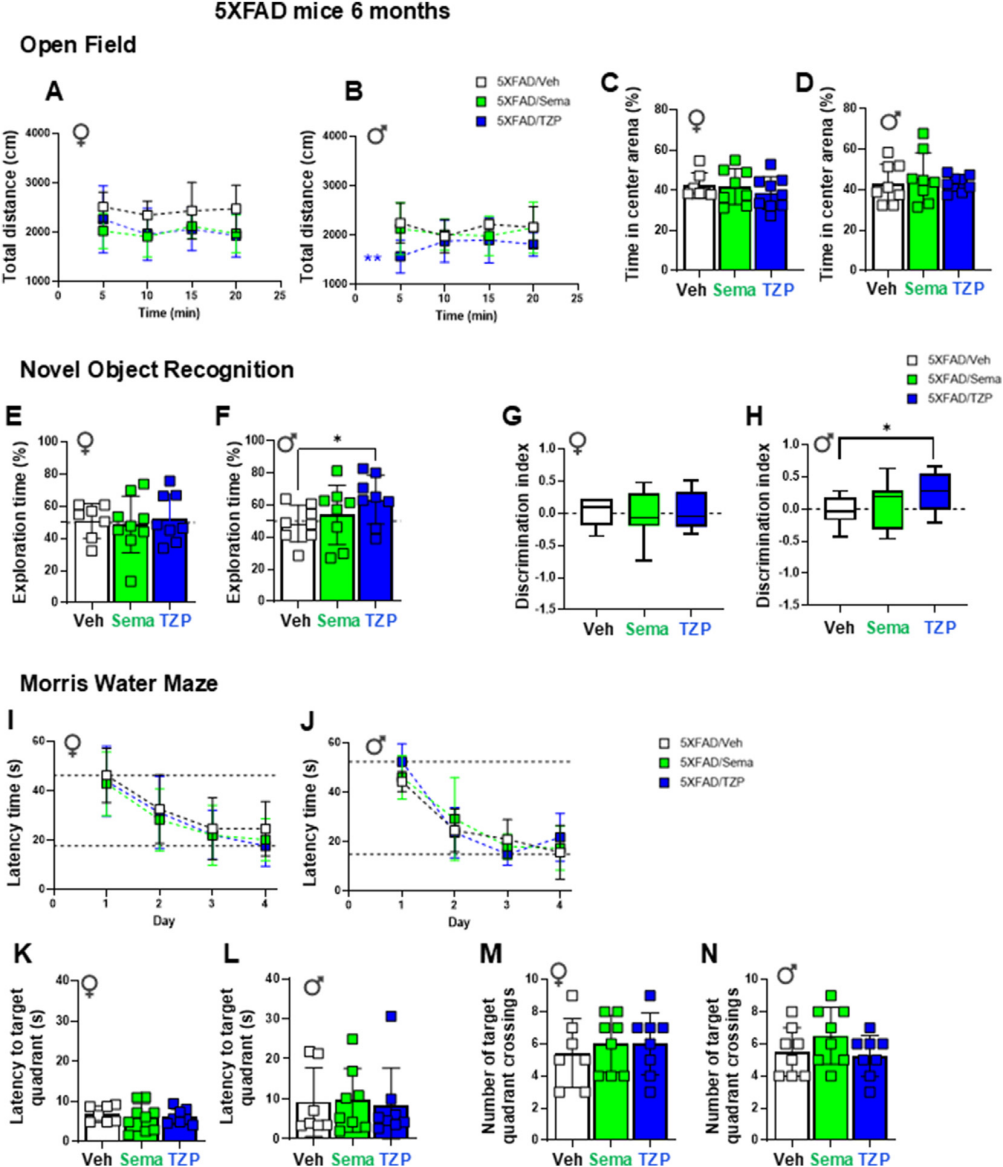
**Memory performance tests in 6-month-old 5XFAD mice.** (A–D) **Open Field Test.** Locomotory activity was evaluated by an Open Field test in 6-month-old female (♀, A) and male (♂, B) 5XFAD mice treated with semaglutide (25 nmol/kg), tirzepatide (10 nmol/kg), or vehicle (saline). The percentage of time spent in the center area of the arena for female (C) and male (D) 5XFAD mice. (E–H) **Novel Object Recognition** tests were performed to assess exploratory behavior and recognition memory. The percentage of exploration time spent on the novel object for 6-month-old female (E) and male (F) 5XFAD mice treated with semaglutide (25 nmol/kg), tirzepatide (10 nmol/kg), or vehicle (saline). The discrimination index, defined by the difference of the time spent exploring the novel object over the total object exploration time, of female (G) and male (H) 5XFAD mice. (I–J) **Morris Water Maze (Training days)**. Daily latency time to reach the platform during the 4-day trial acquisition task (training) of 6-month-old female (I) and male (J) 5XFAD mice treated with semaglutide (25 nmol/kg), tirzepatide (10 nmol/kg), or vehicle (saline). (K–N) **Morris Water Maze (Probe test day).** Latency to the target quadrant (K, L) and number of crossings on the target quadrant (M, N) on Probe test day (Day 5) of 6-month-old female (K, M) and male (L, N) 5XFAD mice treated with semaglutide (25 nmol/kg), tirzepatide (10 nmol/kg), or vehicle (saline). Data for (A, B, I, J) were analyzed by 2-way ANOVA with Sidak's post-hoc test, and for (C–H, K–N) by Mann–Whitney *U* test, or unpaired, two-tailed Student's t test for comparison between vehicle- and semaglutide- or tirzepatide-treated mice. ∗*p* < 0.05, ∗∗*p* < 0.01 vehicle vs. treatment. Data are represented as means ± SD (A-F, I–N), or as box-and-whisker plots (G, H) of female (♀) and male (♂) mice as indicated. Dashed lines in panels I–J indicate the time range (longest and shortest times) required to reach the platform over the 4-day training period. n = 6–10 in each group of vehicle (Veh)-, semaglutide (Sema)-, or tirzepatide (TZP)-treated mice.

Locomotor activity, expressed as total distance travelled and time spent in the center area during the open field test, were not consistently different in 6-month-old male or female 5XFAD mice treated with semaglutide or tirzepatide (Figure 7A–D). On novel object recognition testing, male tirzepatide-treated, but not semaglutide-treated, 5XFAD mice exhibited longer exploration times and higher discrimination indices for the novel object, indicating improved memory (Figure 7E–H). Initial locomotor activity was reduced, but no other behavioral differences were observed in semaglutide- and tirzepatide-treated WT control mice (Supplementary Figure 7A-H). Neither semaglutide nor tirzepatide treatment impacted tasks assessed using the Morris water maze, including latency time, latency to target quadrant, or number of site crossings in target quadrant (Figure 7IN and Supplementary Figure 7I-L). Total distance and swimming speed did not differ among the groups (Supplementary Figure 7M−P).

### 3.8 Semaglutide and tirzepatide do not reduce hippocampal microglial activation or astrogliosis in 6-month-old 5XFAD mice

**Figure 8:**
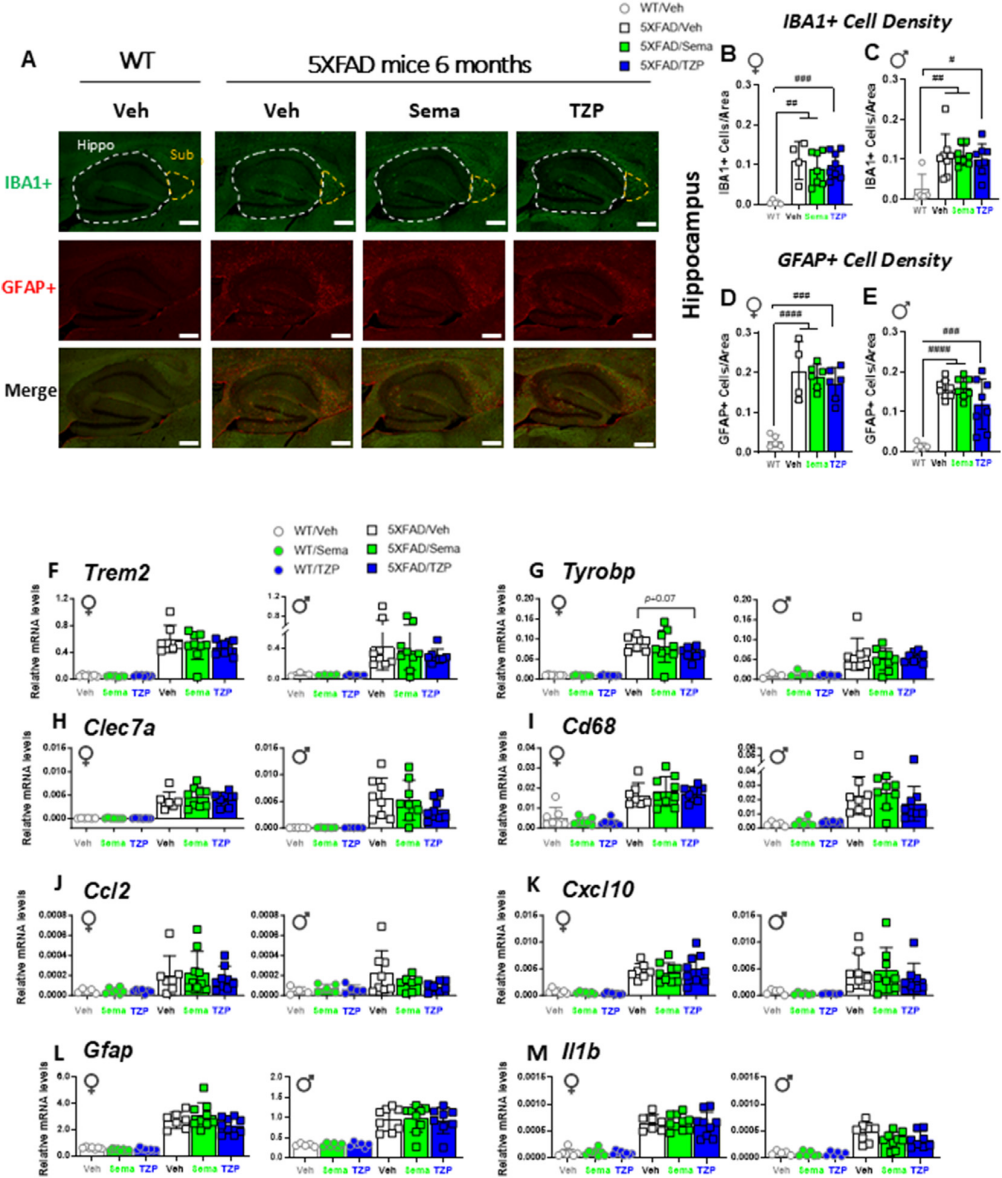
**Semaglutide and tirzepatide do not reduce the number of activated microglia or reactive astroglia, or attenuate dysregulated gene expression related to neurodegeneration in the hippocampus of 6-month-old 5XFAD mice**. (A) Representative images of IBA1+ cells (green), GFAP+ cells (red), or co-stained with both IBA1 and GFAP antibodies (bottom panel) in the hippocampus (Hippo) or subiculum (Sub) of 6-month-old WT and 5XFAD mice treated with semaglutide (25 nmol/kg), tirzepatide (10 nmol/kg), or vehicle (saline). Scale bars represent 300 μm. Areas of interest are encircled by dashed lines. (B–E) Quantification of the number of IBA1+ cells per area (B, C) and the number of GFAP+ cells per area (D, E) in the hippocampus of female (♀) and male (♂) WT and 5XFAD mice. (F–M) Quantitative PCR analysis of transcript levels of *Trem2* (F), *Tyrobp* (G), *Clec7a* (H), *Cd68* (I), *Ccl2* (J), *Cxcl10* (K), *Gfap* (L) and *Il1b* (M) in the hippocampus of 6-month-old female (♀) and male (♂) WT and 5XFAD mice treated with semaglutide (25 nmol/kg), tirzepatide (10 nmol/kg), or vehicle (saline). *Ppia* was used as a reference gene for normalization. Data for (F–M) were analyzed by 2-way ANOVA with Sidak's post-hoc test for comparison of vehicle vs. treatment and for (B–E) by 1-way ANOVA with Tukey's post-hoc test. #*p* < 0.05, ##*p* < 0.01 ###*p* < 0.001, ####*p* < 0.0001 WT vs. 5XFAD mice. Data are represented as means ± SD. n = 3–12 in each group of vehicle (Veh)-, semaglutide (Sema)-, or tirzepatide (TZP)-treated mice.

The extent of microglial activation and reactive astrocytes, as measured by the density of IBA1- and GFAP-positive cells, respectively, were both elevated in the hippocampus (Figure 8A–E) and subiculum (Supplementary Figure 8A-D) of 6-month-old male and female 5XFAD mice compared to WT littermates. However, neither semaglutide nor tirzepatide altered IBA1 or GFAP cell density in either the hippocampus or subiculum of 6-month-old male and female 5XFAD mice (Figure 8A–E, Supplementary Figure 8A-D).

### 3.9 Inflammation-related gene expression is upregulated in the hippocampus of 6-month-old 5XFAD mice

Neuroinflammation is frequently detected in the brain, including within the hippocampal region, of 5XFAD mice with amyloid beta accumulation [25,26]. GLP-1-based medicines, including semaglutide and tirzepatide, exert anti-inflammatory actions in the systemic circulation, peripheral organs, and the brain [[27], [28], [29]]. Accordingly, we investigated the expression of mRNA transcripts encoding biomarkers of inflammation and neurodegeneration in the hippocampus of 6-month-old male and female 5XFAD mice. Levels of mRNA transcripts encoding Triggering Receptor Expressed on Myeloid cell-2 (TREM2), a key regulator that switches myeloid cells from a homeostatic to a neurodegenerative phenotype [29], were upregulated in the hippocampus of male and female 5XFAD mice, but not altered by treatment with semaglutide or tirzepatide (Figure 8F). *Tyrobp*, a gene induced in the earliest stages of microglia activation [30], was upregulated in 5XFAD hippocampal RNA, but its expression was not attenuated by semaglutide treatment. However, *Tyrobp* mRNA levels trended lower in tirzepatide-treated female mice (Figure 8G). Nevertheless, for the majority of mRNA transcripts examined, the expression of genes encoding biomarkers of inflammation such as *Clec7a*, *Cd68*, *Ccl2*, *Cxcl10*, *Gfap*, *Il1b*, *Tnf*, and *Il6* were increased in the hippocampus of 5XFAD vs WT control mice, but not different after treatment with semaglutide or tirzepatide (Figure 8HM, Supplementary Figure 8E-F). In contrast, levels of mRNA transcripts for the GLP-1 and GIP receptors and M2 macrophage markers, including mannose receptor C-type 1 (*Mrc1*), macrophage galactose N-acetyl-galactosamine specific lectin 2 (*Mgl2*) and arginase (*Arg1*), were not elevated in 6-month-old 5XFAD vs WT control mice (Supplementary Figure 8G-K).

## 4 Discussion

We chose to carry out studies initially directed at identification of putative mechanisms linking GLP-1R agonism to attenuation of neurodegeneration using two widely used transgenic mouse models of Alzheimer's disease featuring excessive deposition of amyloid plaques [31]. APP/PS1 mice harbour a mutant form of the Amyloid Precursor Protein (APP), the APP^swe^ mutant and the ΔE9 mutant of PS1 (Presenilin 1). These mice exhibit age-related cognitive impairment and progressive accumulation of beta-amyloid with aging [32]. 5XFAD mice contain the *APP*^swe^ mutation with the Florida (I716V) and London (V717I) mutations of *APP*, together with the M146L and L286V mutations of PS1 [21], resulting in accelerated accumulation of amyloid aggregates, brain pathology, and memory impairment, at a younger age. Our studies examined the effects of GLP-1R agonists in 12-month-old mice, and in younger 6-month-old mice, which likely exhibit less severe neuropathology.

Several previous studies have examined the actions of GLP-1RAs in APP/PS1 mice. Administration of liraglutide, 500 μg/kg/day for 20 weeks reduced amyloid plaque burden and improved cognitive function assessed using object recognition and MWM testing in male and female APP/PS1:db/db mice that were up to 26 weeks of age by the end of the treatment period [33]. Both liraglutide (25 nmol/kg/day) and a dual GIPR-GLP-1R co-agonist DA-JC1(50 nmol/kg/day) reduced Aβ plaque load, decreased astrocyte activation, and attenuated neuroinflammation after a 4 week course of treatment in 10–12-month old female APP/PS1 mice [34]. Similar neuroprotective findings, including improvement in cognition and working memory, were observed after administration of DA5-CH (10 nmol/kg/day), a GIPR-GLP-1R co-agonist, to 9-month-old male and female APP/PS1 mice for 4 weeks [35]. Consistent with these findings, administration of the GIPR-GLP-1R co-agonist DA4-JC (10 nmol/kg/day) for 8 weeks was more effective in reversing memory impairment and decreasing amyloid plaque accumulation and neuroinflammation, relative to findings obtained with equimolar dosing of liraglutide in 9-month-old male and female APP/PS1 mice [27]. Hence, the available data, using treatment regimens and APP/PS1 mice of similar ages to those studied here, demonstrate therapeutic efficacy of GLP-1-based medicines.

Therapeutic efficacy of GLP-1RAs has also been reported in the 5XFAD mouse. NLY01, an exendin-4 derivative, administered using doses of 1 or 10 mg/kg starting at the age of 3 months, for a total of 4 months, resulted in improved spatial learning and attenuated hippocampal amyloid plaque burden and GFAP expression in 5XFAD mice [36]. Administration of liraglutide (25 nmol/kg/day) for 30 days to 3-month-old male and female 5XFAD mice with intracerebroventricular streptozotocin-neuroinflammation resulted in reduction of astrocyte activation and Aβ expression in the cortex and hippocampus [37]. Consistent with these findings, administration of exendin-4 (100 μg/kg twice daily for 16 weeks) improved learning ability, spatial memory, and mitochondrial morphology and attenuated the extent of hippocampal Aβ deposition in 9-month-old male 5XFAD mice [38].

The majority of published studies of GLP-1 medicines in experimental neurodegeneration demonstrate therapeutic activity for GLP-1RAs, including those carried out in mouse models of Alzheimer's disease [39]. In our studies, the doses of semaglutide and tirzepatide administered to APP/PS1 and 5XFAD mice clearly exhibited bioactivity, as evidenced by robust improvements in glucose tolerance and achievement of weight loss. The relatively modest weight loss observed here reflects both the doses selected for tirzepatide and semaglutide, as well as body weight, reflecting the regular chow (and not high fat) diet chosen for the experiments. At the 10 nmol/kg dose employed here, the actions of tirzepatide to produce weight loss are likely mediated through the GLP-1R [40] as tirzepatide is a much weaker agonist at the mouse relative to the human GIPR [41]. Nevertheless, tirzepatide increases insulin sensitivity through weight loss-independent mechanisms, even in *Glp1r*^*−/−*^ mice, at a dose (10 nmol/kg/day) identical to that used in the current studies [40]. There is only limited data on tirzepatide action and dose–response relationships in the context of neurodegeneration that inform our inability to detect therapeutic benefit in the current studies.

Interestingly, we recently showed that tirzepatide, at a dose of 3 nmol/kg, acutely reduces lipopolysaccharide-induced systemic inflammation in both wildtype mice and in mice with neuronal-selective knockout of the GLP-1R, whereas the anti-inflammatory actions of semaglutide are eliminated in mice lacking neuronal GLP-1Rs [28]. Our inability to detect any therapeutic benefit of either semaglutide or tirzepatide in two widely used mouse models of neurodegeneration is puzzling, given multiple reports of therapeutic efficacy of GLP-1RAs in mouse models of neurodegeneration [12,39]. Intriguingly, APP/PS1 mice exhibited greater weight loss with semaglutide relative to that observed in 5XFAD mice, perhaps implying differential CNS sensitivity to GLP-1 medicines in various mouse models of neurodegeneration.

Interestingly, other studies also fail to report positive results with GLP-1RAs in similar mouse models. Hansen et al. were unable to demonstrate therapeutic efficacy of liraglutide, dosed at 100 ng/kg/day or 500 ng/kg/day, in two different APP/PS1 mouse models [42]. Notably, liraglutide had no effect on changes in memory performance or β−amyloid plaque load, when administered for 3 or 5 months [42]. Similarly, long term administration of exendin-4 (500 μg/kg/day for 9 months) failed to reduce Aβ plaque burden or Tau immunoreactivity in 12-month-old 3xTg-AD mice expressing human mutant PS1 (PS1_M146V_), human mutant APP (APP_swe_) and tau (tau _P301L_) protein [43]. More recently, administration of tirzepatide for 8 weeks to 6-month-old APP/PS1 mice at a dose sufficient to reduce blood glucose and modulate CNS gene expression related to glucose transport, had no effect on hippocampal plaque accumulation and did not modify anxiety or cognitive function assessed by novel object recognition and MWM testing [44].

### 4.1 Experimental limitations

Our studies have several limitations. It is possible that the transgenic lines propagated in our animal facility may not exhibit the same extent of neurodegeneration, neuroinflammation and impairment of cognitive function seen when APP/PS1 and 5XFAD mice are housed and studied in other vivariums. Furthermore, the treatment period of 4–5 weeks, as well as the doses of the GLP-1 medicines utilized herein, while sufficient to demonstrate therapeutic efficacy of GLP-1RAs in some studies with APP/PS1 mice [34,35], may not have been sufficient to optimize the neuroprotective properties of semaglutide or tirzepatide when studied here. The effect size of GLP-1 medicines in these mouse models may be modest, requiring greater numbers of mice per experiment to demonstrate therapeutic efficacy. Moreover, we did not examine parameters such as endoplasmic reticulum stress, brain size, neurodegeneration, or mitochondrial activity, hence it is possible that we may have under-estimated the therapeutic potential of semaglutide and tirzepatide. Nevertheless, in our studies, the therapeutic activity of semaglutide and tirzepatide on glucose tolerance and reduction of body weight are dissociated from actions of these agents to reduce amyloid plaque, glial or astrocyte activation, or neuroinflammation or improve memory and behavior.

## CRediT authorship contribution statement

**Leticia Forny Germano:** Writing – original draft, Investigation, Formal analysis. **Jacqueline A. Koehler:** Writing – original draft, Methodology, Investigation, Formal analysis. **Laurie L. Baggio:** Writing – review & editing, Investigation, Formal analysis. **Fiona Cui:** Writing – review & editing, Investigation. **Chi Kin Wong:** Writing – review & editing, Investigation. **Nikolaj Rittig:** Writing – review & editing, Investigation, Formal analysis. **Xiemin Cao:** Writing – review & editing, Investigation. **Dianne Matthews:** Writing – review & editing, Investigation. **Daniel J. Drucker:** Writing – review & editing, Writing – original draft, Supervision, Investigation, Funding acquisition, Conceptualization.

## Declaration of competing interest

Dr. Drucker has served as a consultant or speaker within the past 12 months to Altimmune, Amgen, AstraZeneca Boehringer Ingelheim, Kallyope, Merck Research Laboratories, Novo Nordisk Inc., Pfizer Inc. and Zealand Pharma Inc.

Laurie Baggio is currently an employee of Avalere Health.

None of the authors have anything else to declare.

## Data Availability

Data will be made available on request.
