# Supplementary material for: The GLP-1 medicines semaglutide and tirzepatide do not alter disease-related pathology, behaviour or cognitive function in 5XFAD and APP/PS1 mice
Source: Mol Metab. 2024 Aug 30;89:102019. doi: 10.1016/j.molmet.2024.102019 (PMC11408156; doi:10.1016/j.molmet.2024.102019)
Supplement: Multimedia component 1 [file mmc1.pdf]

Supplementary Figure 1

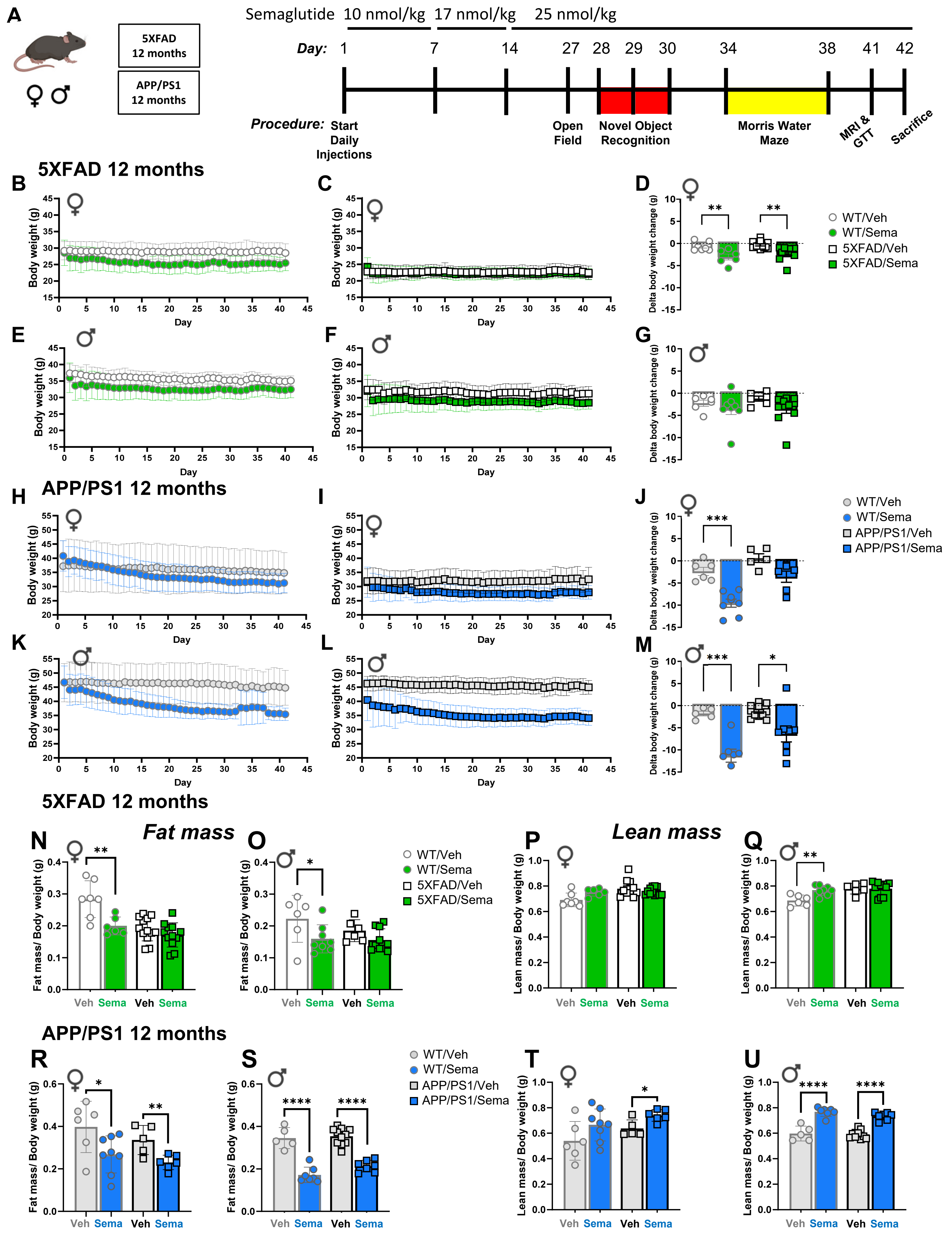

Supplementary Figure 2

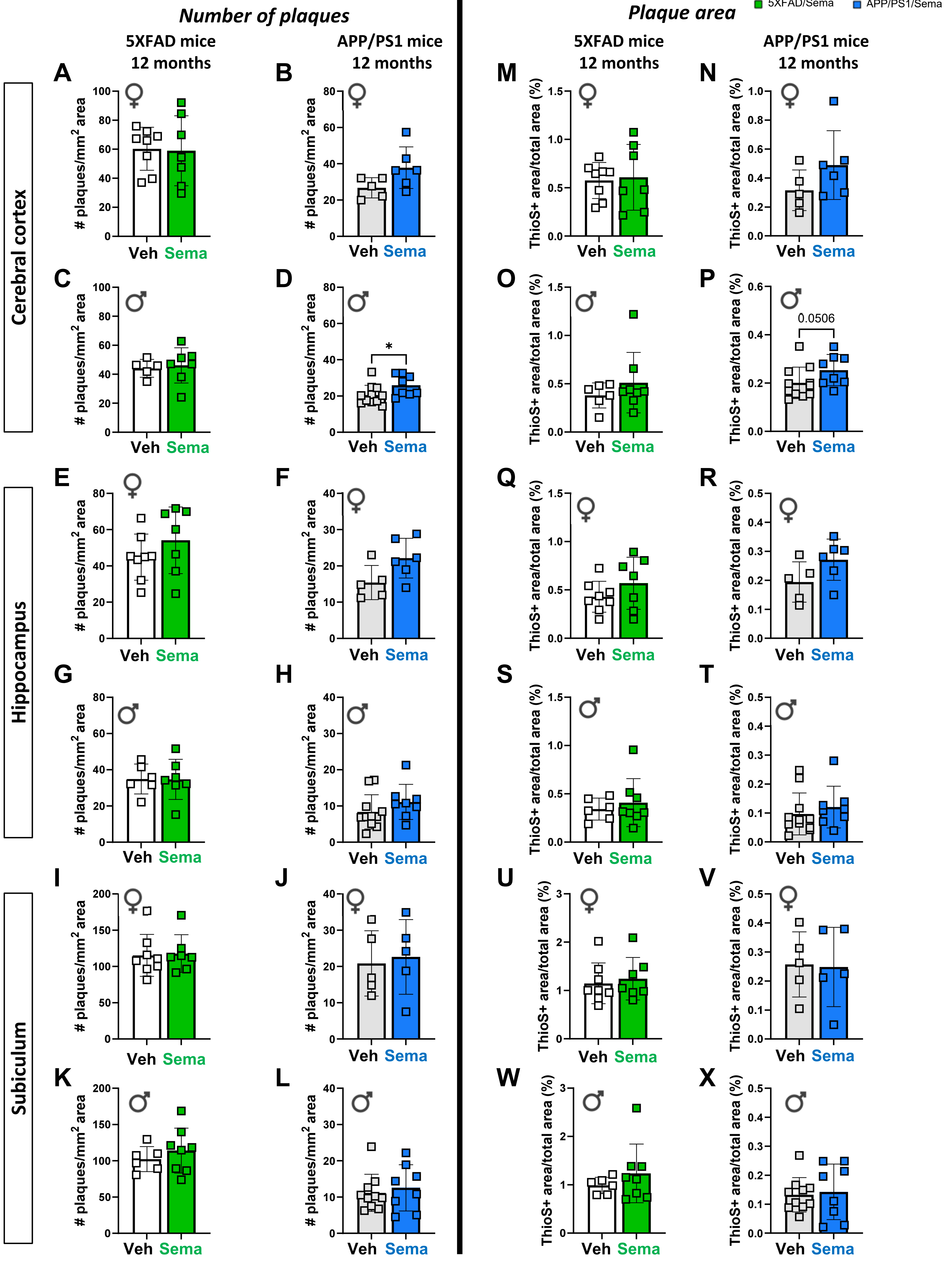

Supplementary Figure 3

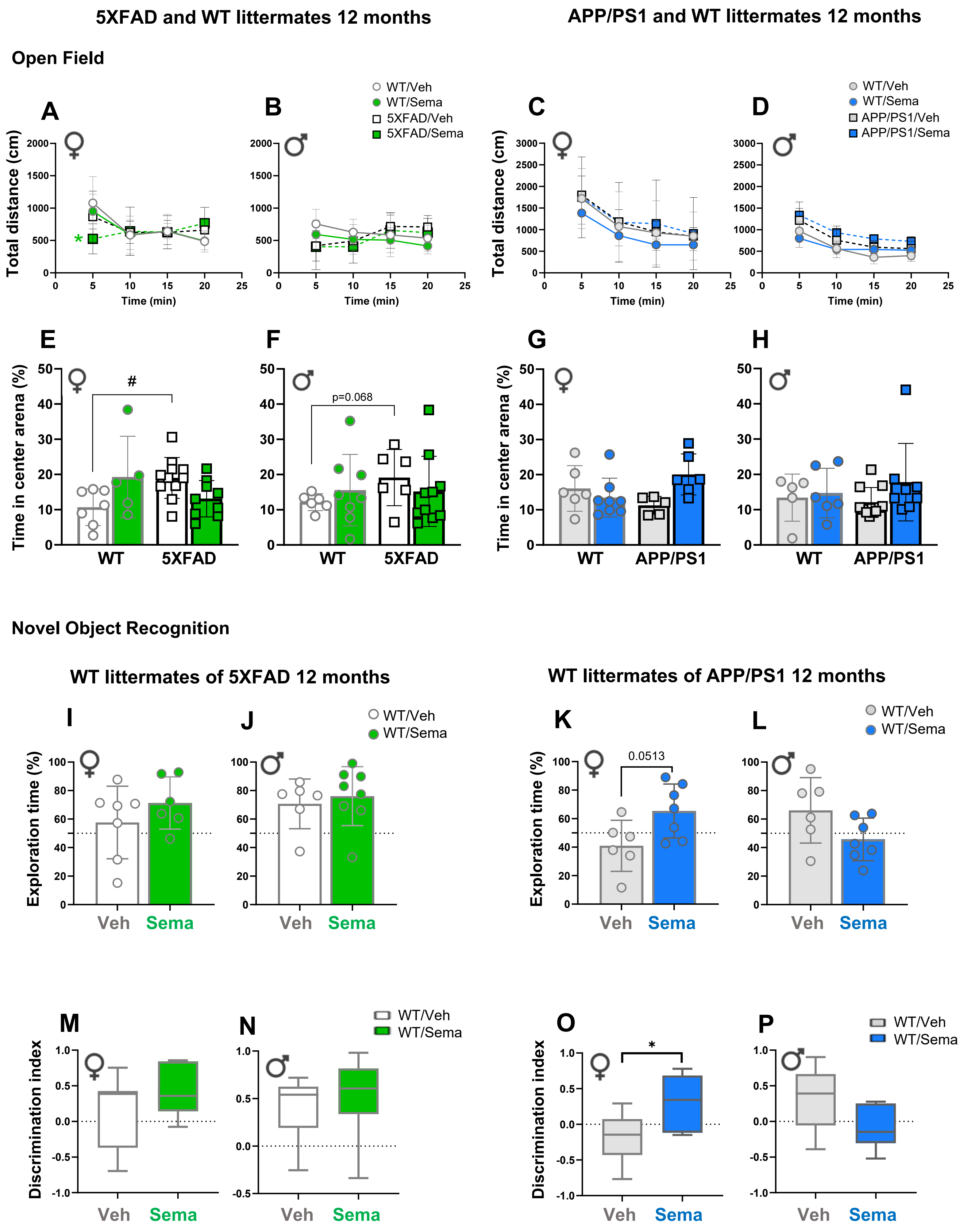

Supplementary Figure 4

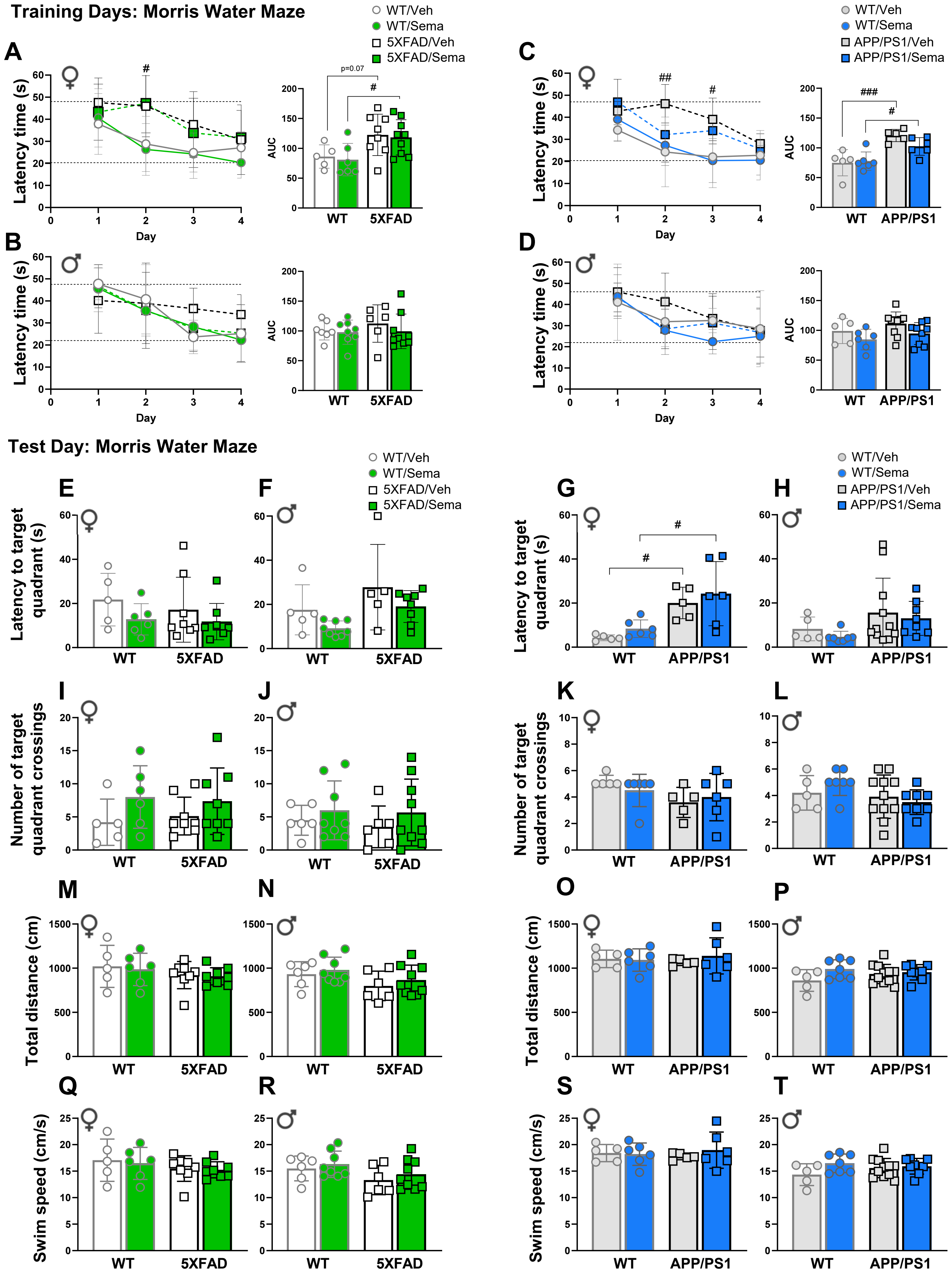

Supplementary Figure 5

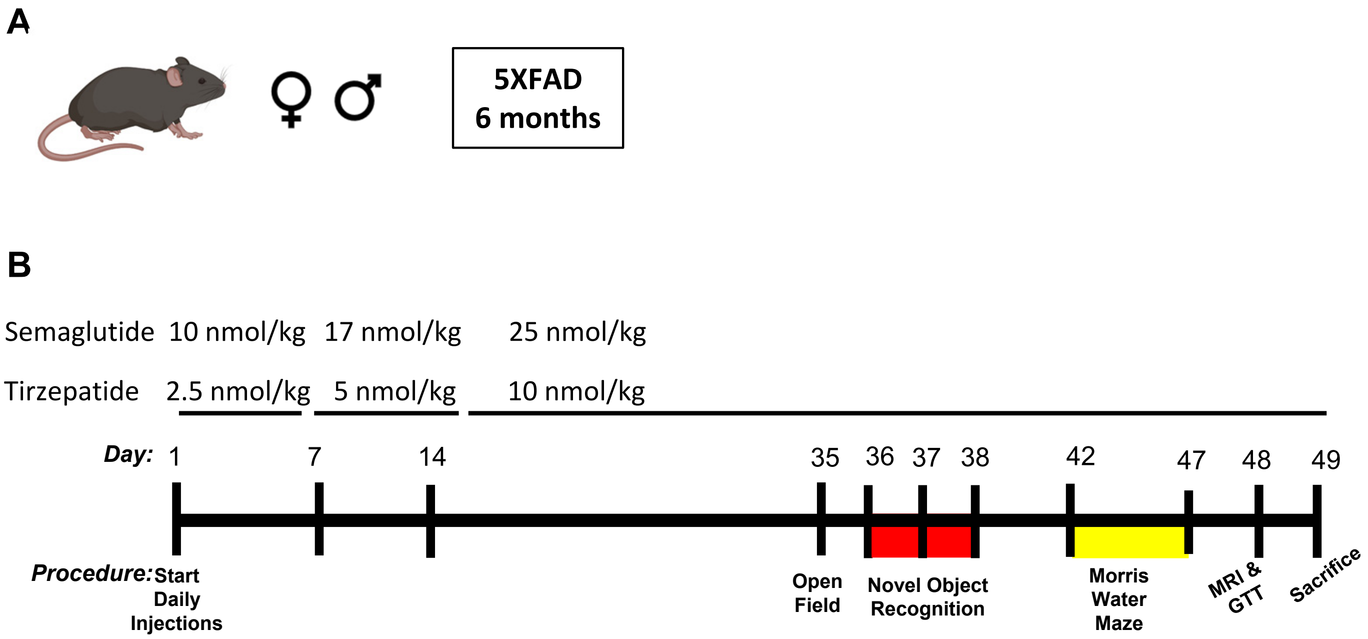

Supplementary Figure 6

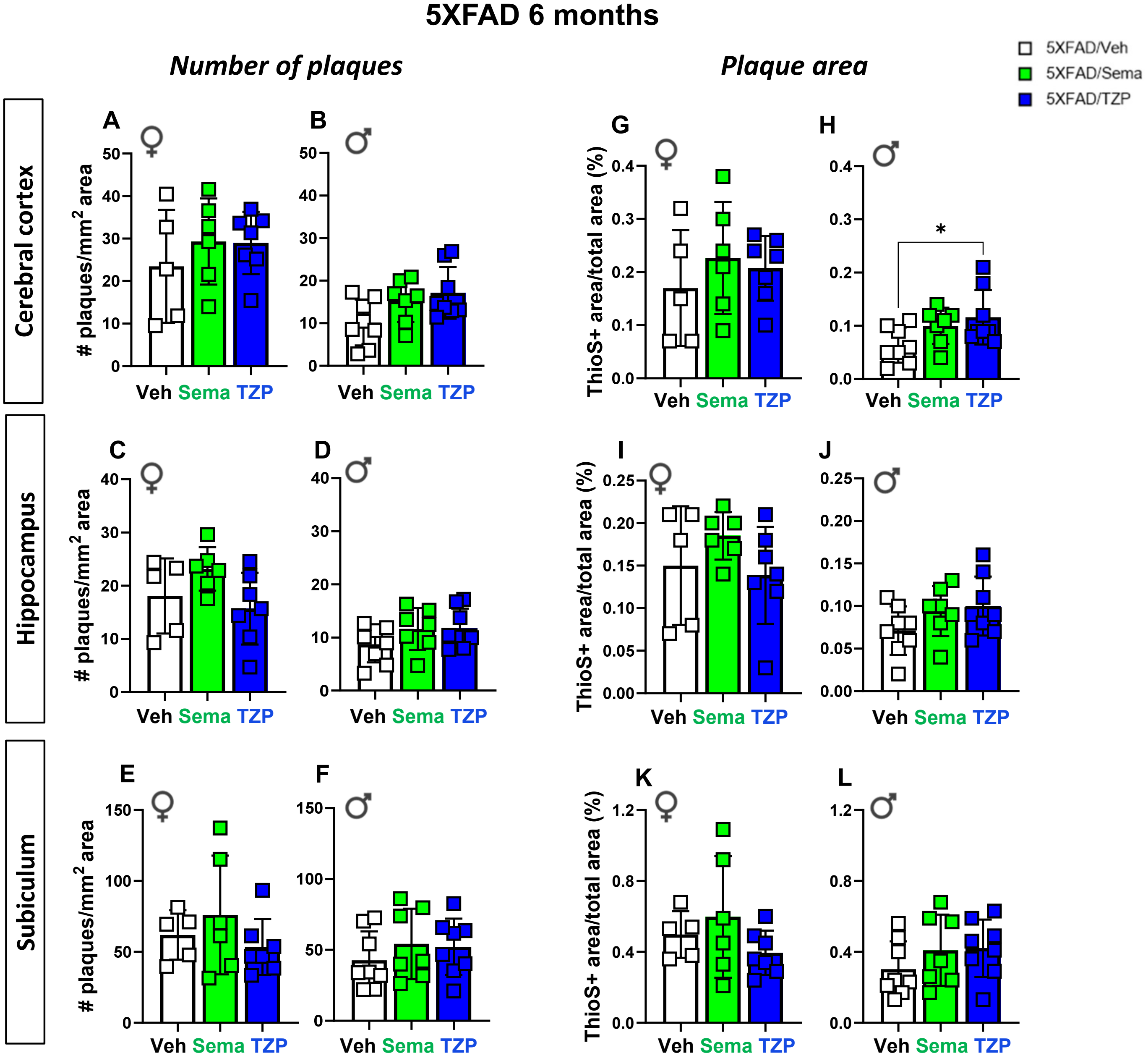

Supplementary Figure 7

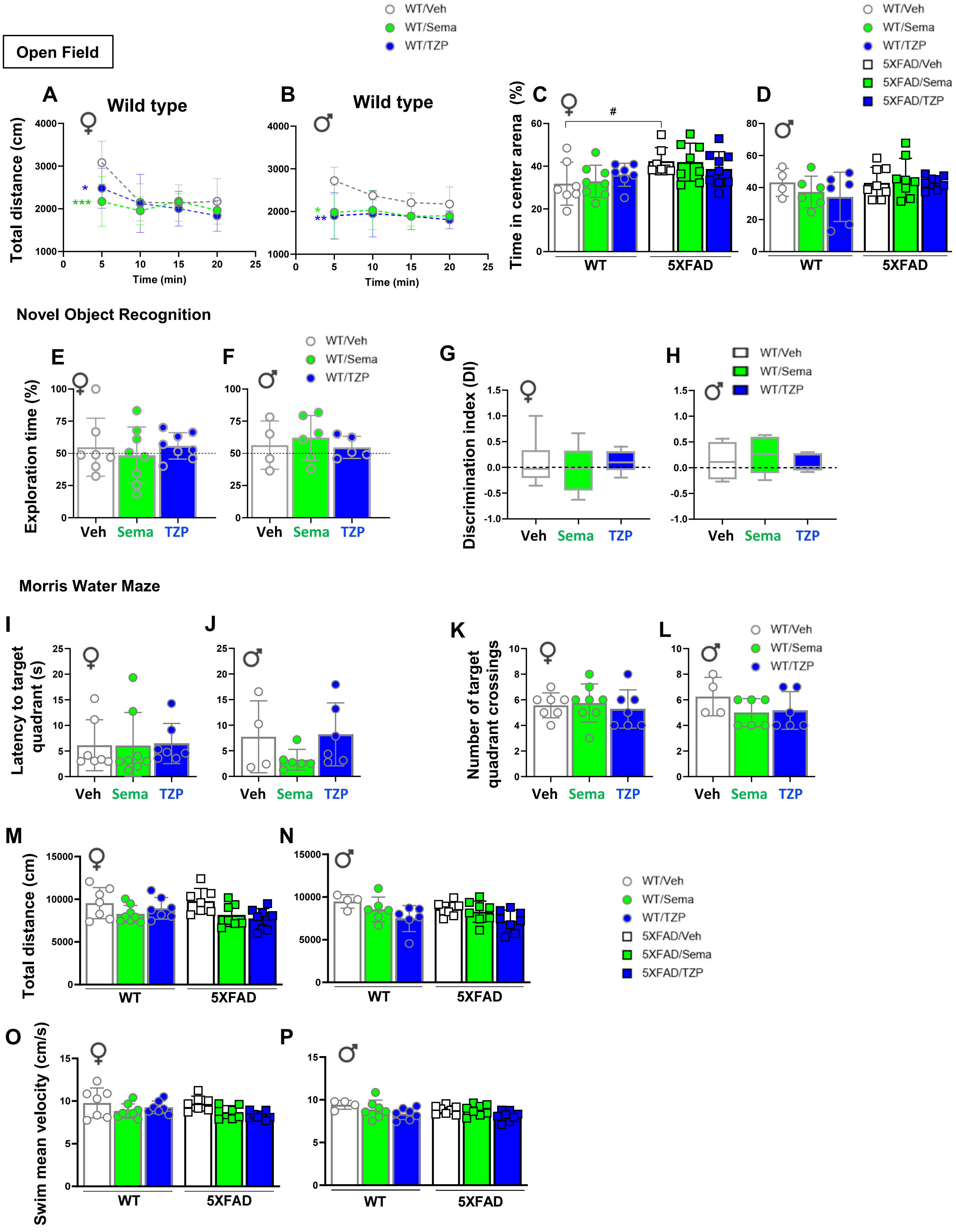

Supplementary Figure 8

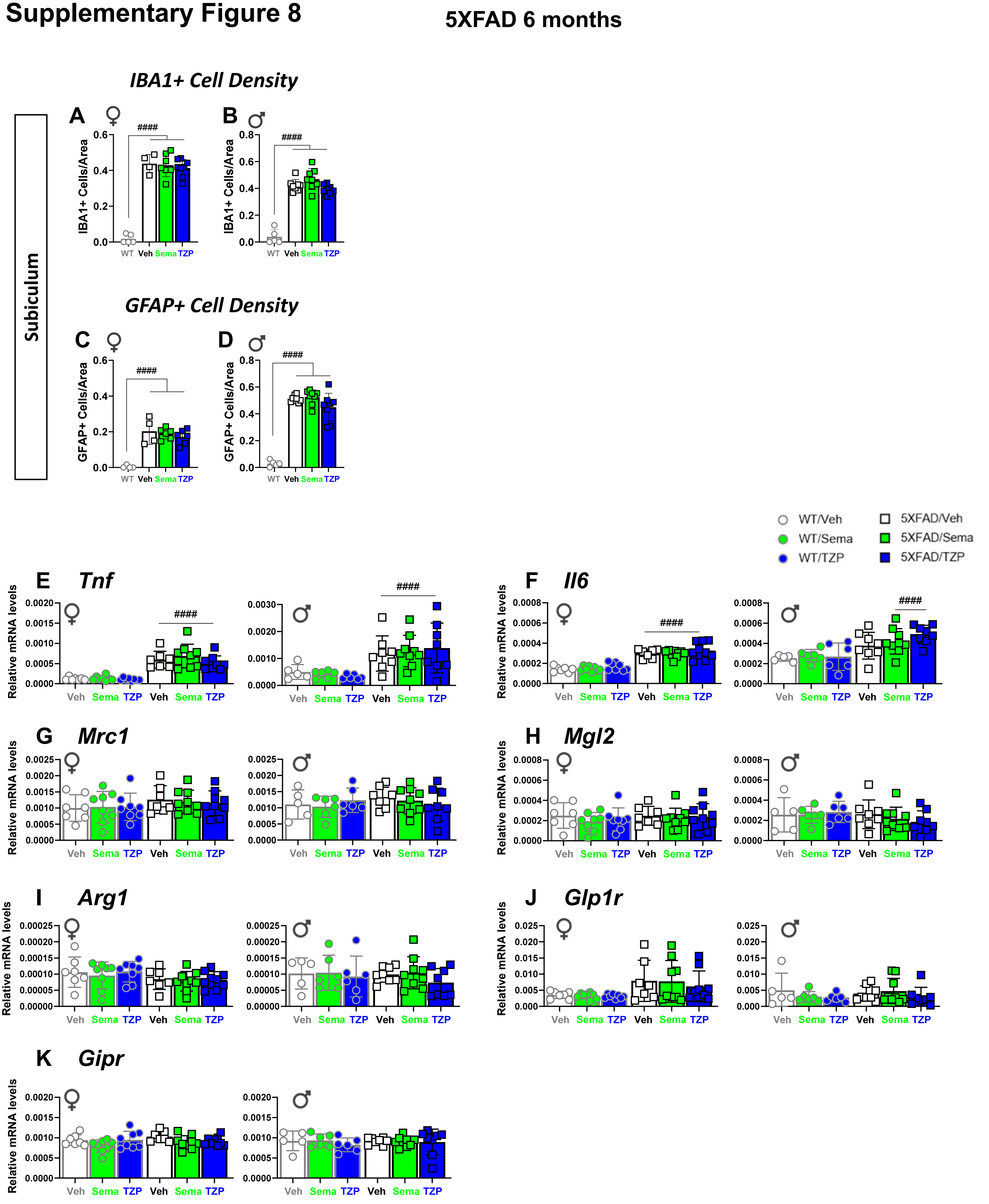

Supplementary Table 1

Catalog number	Symbol	Gene name
Mm01149183_m1	*Tyrobp*	TYRO protein tyrosine kinase binding protein
Mm01253033_m1	*Gfap*	glial fibrillary acidic protein
Mm03047343_m1	*Cd68*	CD68 antigen
Mm00441242_m1	*Ccl2*	chemokine (C-C motif) ligand 2 (MCP1)
Mm04209422_m1	*Trem2*	triggering receptor expressed on myeloid cells 2
Mm00445235_m1	*Cxcl10*	chemokine (C-X-C motif) ligand 10 (IP-10)
Mm01183349_m1	*Clec7a*	C-type lectin domain family 7, member a
Mm00443258_m1	*Tnf*	tumour necrosis factor alpha
Mm00446190_m1	*IL6*	interleukin 6
Mm004344228_m1	*Il1b*	interleukin 1b
Mm01329359_m1	*Mrc1*	mannose receptor C-type 1
Mm00460844_m1	*Mgl2*	macrophage galactose N-acetyl-galactosamine specific lectin 2
Mm00475988_m1	*Arg1*	arginase
Mm00445292_m1	*Glp1r*	glucagon-like peptide 1 receptor
Mm01316344_m1	*Gipr*	gastric inhibitory polypeptide receptor
Mm02342430_g1	*Ppia*	Cyclophilin

Supplemental Figures and Table

Supplementary Figure 1: Experimental timeline, daily body weight and body composition after treatment of 12-month-old 5XFAD and APP/PS1 mice. (A) Female and male 5XFAD, APP/PS1 and aged-matched wildtype littermate mice were 11-12 months of age at the beginning of the treatment. The semaglutide dose regimen was 10 nmol/kg for 7 days, 17 nmol/kg for 7 days and 25 nmol/kg for the remainder of the study. Behavior tests began on Day 27. After 3 days of rest, EchoMRI measurements and i.p. glucose tolerance tests (GTT) were performed. After 6 weeks of treatment (Day 42), the mice were terminated. Daily body weight of matched WT controls (B, E) with 5XFAD (C, F), and matched WT controls (H, K) with APP/PS1 (I, L) female (♀) and male (♂) mice as indicated. Change in body weight change (final minus initial body weight) of regular chow diet-fed WT control and 5XFAD (D, G), and APP/PS1 (J, M) mice. Fat mass normalized to body weight on Day 41 of 5XFAD (N, O), APP/PS1 (R, S) and lean mass normalized to body weight of 5XFAD (P, Q), APP/PS1 (T,U) and WT-matched female control (♀) and male (♂) mice treated with semaglutide (25 nmol/kg) or vehicle (saline). Data were analyzed by 2-way ANOVA with Sidak's post-hoc test for comparison of WT control or transgenic vehicle- vs. semaglutide-treated mice. * $p < 0.05$, ** $p < 0.01$, *** $p < 0.001$, **** $p < 0.0001$ vehicle vs. semaglutide-treated mice. Data are represented as means ± SD. n=5-13 in each group of vehicle- (Veh), or semaglutide- (Sema) treated mice.

Supplementary Figure 2: Semaglutide does not alter the number of plaques or amyloid plaque area in 12-month-old 5XFAD and APP/PS1 mice. (A-L) Quantification of the number of ThioS+ amyloid plaques per area in the cerebral cortex (A-D), hippocampus (E-H), and subiculum (I-L) after Thioflavin S staining in 12-month-old 5XFAD (left panels) and APP/PS1 (right panels) female (♀) and male (♂) mice treated with semaglutide (25 nmol/kg) or vehicle (saline). (M-X) Quantification of ThioS+ area in the cerebral cortex (M-P), hippocampus (Q-T), and subiculum (U-X) after Thioflavin S staining in 12-month-old 5XFAD (left panels) and APP/PS1 (right panels) of female (♀) and male (♂) mice as indicated. Data were analyzed by Mann-Whitney *U* test, or unpaired, two-tailed Student's t test. * $p < 0.05$ vehicle- vs. semaglutide-treated mice. Data are represented means ± SD. n=5-11 in each group of vehicle- (Veh), or semaglutide- (Sema) treated mice.

Supplementary Figure 3: Locomotory and exploration activity of 12-month-old 5XFAD, APP/PS1, and wildtype (WT) littermate mice. (A-H) **Open Field test.** Locomotory activity during the 20 minutes in the open field arena of 12-month-old 5XFAD (A, B), APP/PS1 (C, D), and WT matched female (♀) and male (♂) mice treated with semaglutide (25 nmol/kg) or vehicle (saline). The percentage of time spent in the center area of the arena of 5XFAD (E, F), APP/PS1 (G, H), and WT littermates. (I-P) **Novel Object Recognition test**. The percentage of exploration time spent on the novel object of vehicle or semaglutide (25 nmol/kg)-treated WT littermates of 5XFAD (I, J) and APP/PS1 (K, L) mice. The discrimination index , defined by the time spent investigating the novel object over the total object exploration time, in WT littermates of 5XFAD (M, N) and APP/PS1 (O, P) mice. Data for (A-H) were analyzed by 2-way ANOVA with Sidak's post-hoc test comparison, and for (I-P) by Mann-Whitney *U* test, or unpaired, two-tailed Student's t test. * $p < 0.05$ vehicle- vs. semaglutide-treated mice. # $p < 0.05$ WT vs. transgenic mice. Data are represented as means ± SD (A-L), or as box-and-whisker plots (M-P) of female (♀) and male (♂) mice as indicated. n=5-8 in each group of vehicle- (Veh) or semaglutide- (Sema) treated mice.

Supplementary Figure 4: Morris Water Maze performance of 12-month-old 5XFAD, APP/PS1 and wildtype (WT) control mice. (A-D) **Training days**. *Left panels:* Daily latency time to reach the platform during the 4-day trial acquisition task (training) for vehicle or semaglutide (25 nmol/kg)-treated 5XFAD (A, B), APP/PS1 (C, D) and matched WT female (♀) and male (♂) mice. *Right panels:* Area Under the Curve (AUC) of the latency times over the 4-day training period. (E-T) **Probe test day**. Latency time to the target quadrant on Probe test day (Day 5) for vehicle- or semaglutide (25 nmol/kg)-treated 5XFAD (E, F), APP/PS1 (G, H), and WT littermates. The number of crossings on the target quadrant for vehicle- or semaglutide (25 nmol/kg)-treated 5XFAD (I, J), APP/PS1 (K, L), and WT matched mice. The total distance traveled by 5XFAD (M, N), APP/PS1 (O, P), and WT littermate mice treated with semaglutide (25 nmol/kg) or vehicle (saline). Swimming speed of 5XFAD (Q, R), APP/PS1 (S, T) and WT littermate mice treated with semaglutide (25 nmol/kg) or vehicle (saline). Data were analyzed by 2-way ANOVA with Sidak's post-hoc test. # $p < 0.05$, ## $p < 0.01$ WT vs. transgenic mice. Data are represented as means ± SD of female (♀) and male (♂) mice as indicated. Dashed lines in panels A-D indicate the time range (longest and shortest time) required to reach the platform over the 4-day training period. n=5-10 in each group of vehicle- (Veh) or semaglutide- (Sema) treated mice.

Supplementary Figure 5: Experimental timeline for dose escalation of drugs and behavioral tests for 6-month-old 5XFAD mice. (A) Female (♀) and male (♂) 5XFAD and matched WT littermate mice were 6 months old at the beginning of the treatment. (B) The drug doses were escalated to avoid side effects and higher weight loss. The semaglutide dose regimen was 10 nmol/kg for 7 days, 17 nmol/kg for 7 days, and 25 nmol/kg for the remainder of the study. For tirzepatide the doses were 2.5 nmol/kg, 5 nmol/kg and 10 nmol/kg. Behavior tests began on Day 35. After 3 days of rest, EchoMRI measurements and i.p. glucose tolerance tests (GTT) were performed the day before the end of the study. After 7 weeks of treatment (Day 49), the mice were terminated.

Supplementary Figure 6: Impact of tirzepatide and semaglutide therapy on the number or area of amyloid plaques in 6-month-old 5XFAD mice. (A-F) Quantification of the number of ThioS+ amyloid plaques per area in the cerebral cortex (A, B), hippocampus (C, D), and subiculum (E, F) after Thioflavin S staining in 6-month-old female (♀) and male (♂) 5XFAD mice treated with semaglutide (25 nmol/kg), tirzepatide (10 nmol/kg), or vehicle (saline). (G-L) Quantification of ThioS+ area in the cerebral cortex (G, H), hippocampus (I, J), and subiculum (K, L) after Thioflavin S staining in 6-month-old 5XFAD mice treated with semaglutide (25 nmol/kg), tirzepatide (10 nmol/kg), or vehicle (saline). Data were analyzed by 1-way ANOVA with Tukey's post-hoc test * $p < 0.05$ vehicle vs. treatment. Data are represented as means ± SD. n=5-8 in each group of vehicle- (Veh), semaglutide- (Sema), or tirzepatide- (TZP) treated mice.

Supplementary Figure 7: Semaglutide and tirzepatide do not affect exploration activity, or Morris water maze performance in 6-month-old 5XFAD or wildtype(WT) littermate mice. (A-D) **Open Field Test.** Locomotory activity during an Open Field test of 6-month-old female (♀, A) and male (♂, B) WT littermates of 5XFAD mice treated with semaglutide (25 nmol/kg), tirzepatide (10 nmol/kg), or vehicle (saline). The percentage of time spent in center area of the arena of vehicle-, semaglutide (25 nmol/kg)-, or tirzepatide (10 nmol/kg)-treated 5XFAD and wildtype matched female (C) and male (D) mice. (E-H) **Novel Object Recognition** test was performed to assess exploratory behavior and recognition memory. The percentage of exploration time spent on the novel object for female (E) and male (F) WT littermates of 5XFAD mice treated with semaglutide (25 nmol/kg), tirzepatide (10 nmol/kg), or vehicle (saline). The discrimination index , defined by the difference in the time spent exploring the novel object over

the total object exploration time, for female (G) and male (H) WT littermates of 5XFAD mice. (I-P) **Probe test day of Morris Water Maze**. Latency to the target quadrant on Probe test day (Day 5) for female (I) and male (J) WT littermates of 5XFAD mice and the number of crossings on the target quadrant for WT littermate female (K) and male (L) mice treated with semaglutide (25 nmol/kg), tirzepatide (10 nmol/kg), or vehicle (saline). The total distance traveled by 6-month-old vehicle-, semaglutide (25 nmol/kg)-, or tirzepatide (10 nmol/kg)-treated 5XFAD and WT littermate female (M) and male (N) mice. Swimming speed of vehicle-, semaglutide (25 nmol/kg)-, or tirzepatide (10 nmol/kg)-treated 5XFAD and WT littermate female (O) and male (P) mice. Data for (A-D, M-P) were analyzed by 2-way ANOVA for comparison of vehicle vs. treatment, or WT vs. transgenic mice, and (E-L) by 1-way ANOVA with Tukey's post-hoc test. * $p < 0.05$, *** $p < 0.001$ vehicle vs. treatment, # $p < 0.05$ WT vs. transgenic mice. Data are represented as means ± SD (A-F, I-P), or as box-and-whisker plots (G,H) of female (♀) and male (♂) mice as indicated. Dashed lines in panels I-J indicate the time range (longest and shortest times) required to reach the platform for each group of mice over the 4-day training period. n=5-12 in each group of vehicle- (Veh), semaglutide- (Sema), or tirzepatide- (TZP) treated mice.

Supplementary Figure 8: Semaglutide and tirzepatide do not reduce the number of activated microglia or astrogliosis or improve dysregulated gene expression related to inflammation in the hippocampus of 6-month-old 5XFAD mice. (A-D) Quantification of the number of IBA1+ cells per area (A, B) and the number of GFAP+ cells per area (C, D) in the subiculum of 6-month-old WT and 5XFAD mice treated with semaglutide (25 nmol/kg), tirzepatide (10 nmol/kg), or vehicle (saline). Quantitative PCR analysis of transcript levels of *Tnf* (E), *Il6* (F), *Mrc1*(G), *Mgl2* (H), *Argl* (I), *Glp1r* (J) and *Gipr* (K) in the hippocampus of 6-month-old female and male WT and 5XFAD mice treated with semaglutide (25 nmol/kg), tirzepatide (10 nmol/kg), or vehicle (saline). *Ppia* was used as a reference gene for normalization. Data for (E-K) were analyzed by 2-way ANOVA with Sidak's post-hoc test for comparison of vehicle vs. treatment and for (A-D) by 1-way ANOVA with Tukey's post-hoc test. #### $p < 0.0001$ WT vs. 5XFAD mice. Data are represented as means ± SD of female (♀) and male (♂) mice as indicated. n=3-12 in each group of vehicle- (Veh), semaglutide- (Sema), or tirzepatide- (TZP) treated mice.

Supplementary Table 1

List and identity of TaqMan PCR probes
